# Introduction of Deep Learning in Thermographic Monitoring of Cultural Heritage and Improvement by Automatic Thermogram Pre-Processing Algorithms

**DOI:** 10.3390/s21030750

**Published:** 2021-01-22

**Authors:** Iván Garrido, Jorge Erazo-Aux, Susana Lagüela, Stefano Sfarra, Clemente Ibarra-Castanedo, Elena Pivarčiová, Gianfranco Gargiulo, Xavier Maldague, Pedro Arias

**Affiliations:** 1 GeoTECH Group, CINTECX, Universidade de Vigo, 36310 Vigo, Spain; ivgarrido@uvigo.es (I.G.); parias@uvigo.es (P.A.); 2 Escuela de Ingeniería Eléctrica y Electrónica, Universidad del Valle, Cali 760032, VA, Colombia; jorge.erazo@correounivalle.edu.co or; 3 Facultad de Ingeniería, Institución Universitaria Antonio José Camacho, Cali 760046, VA, Colombia; 4 Department of Cartographic and Terrain Engineering, University of Salamanca, Calle Hornos Caleros, 50, 05003 Ávila, Spain; sulaguela@usal.es; 5 Department of Industrial and Information Engineering and Economics (DIIIE), University of L’Aquila, Piazzale E. Pontieri 1, Monteluco di Roio-, I-67100 L’Aquila (AQ), Italy; 6 Computer Vision and Systems Laboratory, Department of Electrical and Computer Engineering, Université Laval, 1065, av. de la Médecine, Québec, QC G1V 0A6, Canada; clemente.ibarra-castanedo@gel.ulaval.ca (C.I.-C.); xavier.maldague@gel.ulaval.ca (X.M.); 7 Faculty of Technology, Technical University in Zvolen, Ul. T.G. Masaryka 2117/24, 960 01 Zvolen, Slovakia; pivarciova@tuzvo.sk; 8 Individual Company of Restoration (Gianfranco Gargiulo), Via Tiberio 7b, I-80073 Capri (NA), Italy; gianfrancogargiulo79@gmail.com

**Keywords:** infrared thermography, deep learning, mask R-CNN, thermal principles, cultural heritage, preservation, monitoring, marquetry, automation

## Abstract

The monitoring of heritage objects is necessary due to their continuous deterioration over time. Therefore, the joint use of the most up-to-date inspection techniques with the most innovative data processing algorithms plays an important role to apply the required prevention and conservation tasks in each case study. InfraRed Thermography (IRT) is one of the most used Non-Destructive Testing (NDT) techniques in the cultural heritage field due to its advantages in the analysis of delicate objects (i.e., undisturbed, non-contact and fast inspection of large surfaces) and its continuous evolution in both the acquisition and the processing of the data acquired. Despite the good qualitative and quantitative results obtained so far, the lack of automation in the IRT data interpretation predominates, with few automatic analyses that are limited to specific conditions and the technology of the thermographic camera. Deep Learning (DL) is a data processor with a versatile solution for highly automated analysis. Then, this paper introduces the latest state-of-the-art DL model for instance segmentation, Mask Region-Convolution Neural Network (Mask R-CNN), for the automatic detection and segmentation of the position and area of different surface and subsurface defects, respectively, in two different artistic objects belonging to the same family: Marquetry. For that, active IRT experiments are applied to each marquetry. The thermal image sequences acquired are used as input dataset in the Mask R-CNN learning process. Previously, two automatic thermal image pre-processing algorithms based on thermal fundamentals are applied to the acquired data in order to improve the contrast between defective and sound areas. Good detection and segmentation results are obtained regarding state-of-the-art IRT data processing algorithms, which experience difficulty in identifying the deepest defects in the tests. In addition, the performance of the Mask R-CNN is improved by the prior application of the proposed pre-processing algorithms.

## 1. Introduction

Preventive and conservation interventions in cultural heritage are essential tasks to protect objects of incalculable human value. People’s concern for the cultural heritage protection goes back several centuries, with the first legislation to protect monuments and works of art appearing in Europe during the 15th century [1]. Heritage objects provide cultural, spiritual, and aesthetic satisfaction, and economic benefits both in terms of cultural consumption and increased employment and income, in addition to being indicators of the course of human life [2,3].

Therefore, the use of the most up-to-date technologies and the corresponding most advanced data processing algorithms is necessary to apply the required preventive and conservation tasks in each specific case under analysis, facing the inevitable object deterioration due to the passage of time. Specifically, inspection technologies must be able to avoid producing new defects in the whole 3D structure, and (their algorithms) to identify defects from their initial growth phase, in order to implement prevention tasks. In addition, both technologies and algorithms should be able to identify the most damaged parts in order to implement conservation tasks. In case of a late, wrong, or absent intervention, the damage can be irreversible by leading to an anticipated degradation of the artistic object.

### 1.1. InfraRed Thermography (IRT) within Cultural Heritage

Currently, InfraRed Thermography (IRT) is defined as one of the most attractive inspection technologies within cultural heritage [4]. IRT presents the advantageous features that correspond to a Non-Destructive Testing (NDT) technique, i.e., (i) non-intrusion and non-damage to the integrity of the object as opposed to destructive techniques, and (ii) higher objectivity and speed compared to traditional methods [5]. In addition, IRT presents added advantageous features such as: (i) The non-contact with the heritage object, (ii) the real time operation, very important for cultural heritage monitoring, (iii) the ability to analyze any surface regardless of the type of object, (iv) the possibility to monitor many points of an object at the same time, (v) the capacity to perform large-scale studies of objects, (vi) the interpretation of the results in two and three dimensions [6,7], and (vii) the possibility of a qualitative and quantitative analysis [8,9].

IRT is based on the measurement of the radiation emitted by an object in one of the sub-bands of the InfraRed (IR) spectrum. In cultural heritage, the emitted radiation is measured by an array of sensors installed inside a camera (called IR camera). These sensors are sensitive to either: (i) The Long-Wave InfraRed sub-band (LWIR, with a wavelength range from 7 µm to 14 µm), or (ii) the Medium-Wave InfraRed sub-band (MWIR, with a wavelength range from 3 µm to 5 µm) [10,11]. Then, the radiation measured by each sensor is converted into temperature as output data of the IR camera: Radiation is related to temperature through the Stefan-Boltzmann law [12]. In this way: (i) The lens of the IR camera is not in contact with the object during the inspection, (ii) the radiation emitted by the object within the LWIR or MWIR sub-band is measured by the IR camera, converting it to temperature values in real time, and (iii) a thermal image or a sequence of thermal images is obtained as final result [13], in which each pixel represents the temperature value calculated in each of the sensors of the IR camera.

Therefore, a temperature map of an artistic object in one or more instants during a monitoring campaign can be obtained by IRT. This temperature map represents the thermal behavior of the object, taking into account: (i) Its surface (passive IRT), or (ii) its surface and its shallower inner layer (a few centimeters, e.g., maximum of 1.5 cm [14] and 5 cm [15] for plaster and reinforced concrete objects, respectively), in case of applying a more intense thermal excitation than that generated by the solar radiation to the object by artificial heat sources (active IRT) [16]. Moreover, the thermal behavior of a defect is different with respect to the thermal behavior of its unaltered surroundings in the object under inspection. The reason is because the defects have different values in their thermophysical properties compared to the thermophysical properties of the unaltered volume of the object regardless of the type of defect [17,18]. Thus, the defect position and the defect area can be identified and determined, respectively, by searching for anomalous temperature values through the study of the relative pixel values of the thermal images acquired (qualitative analysis). In case of depth estimation and thermal characterization of the defect, and even thermal characterization of the object, the study on the thermal images is rather an analysis of the pixel values individually instead of a comparison among them (quantitative analysis) [19].

Although the first IR cameras on the market date from 1965 [20], IRT can still be considered an up-to-date inspection technology due to the continuous evolution of this technology, including within the cultural heritage field. This evolution concerns both IRT data acquisition methods, such as the continuous improvement of the IR cameras and the new forms of thermal excitation (such as induced eddy current, and microwave and ultrasound excitation), and IRT data processing algorithms. Proof of this are the reviews by Garrido et al. [10,11] that compile the latest advances in IRT data acquisition methods and IRT data processing algorithms when inspecting an infrastructure, including heritage objects, respectively.

#### 1.1.1. IRT Data Processing Algorithms Used within Cultural Heritage (Related Work)

IRT data processing algorithms are needed because raw thermal images usually present excessive noise that prevents a good qualitative/quantitative study. Such noise can be caused by one or more of the following phenomena that typically occur on the surface of an object: (i) Reflection, (ii) shading, and (iii) non-uniform heating or cooling. In addition, noise is also caused by the IR camera: (i) Vignetting, (ii) spectrometer drift, (iii) radial distortion, and (iv) fixed pattern noise [21,22]. Therefore, data processing algorithms applied to thermal images are necessary to mitigate noise and thus maintain the advantages of IRT described in the previous section.

There are IRT data processing algorithms based on either thermal-based analysis and physics of heat transfer, or mathematics and analytical procedures within cultural heritage. In the first group of algorithms, Pixelwise Algorithm for Time-Derivative of Temperature (PATDT), Partial Least Square Thermography (PLST), Pulse Phase Thermography (PPT), and Thermography Signal Reconstruction (TSR) are the most advanced algorithms. Using PATDT, Yao et al. [23] detected the position of several defects with different depths simulating detachments in a panel painting made of poplar wood, *Madonna* specimen. PATDT simplifies the defect detection by summarizing the thermal image sequence under study into one single image. With PLST, Zhang et al. [24] revealed the position of subsurface defects in two oil paintings with different canvas supports (one made of hemp and nettle, and the other made of flax and juniper), representing the James Abbott McNeill Whistler’s *Portrait of the Painter’s Mother*. Finally, Ibarra-Castanedo et al. [25] and Sfarra et al. [26] identified the position of subsurface defects present in the object studied in [23] and beneath decorative surface coatings applying PPT and TSR, respectively.

As for mathematical and analytical algorithms, the most advanced algorithms are Principal Component Thermography (PCT) and its improved versions (Sparse Principal Component Thermography (SPCT) and Independent Component Thermography (ICT)), and Dynamic Thermal Tomography (DTT). Thickett et al. [27] applied PCT to a series of mediaeval Limoges enamel plaques from the Werner Collection, displayed at Rangers House, London. PCT results shown the position of cracks with different depths. Using SPCT and DTT, Sfarra et al. [13] and Vavilov et al. [28] identified the position of a buried window covered by bricks and plaster over time and three zones of embedded beams underneath belonging to a heritage bell tower, and revealed subsurface features in ancient wall frescos, respectively. Finally, Yao et al. [23] also detected the position of the subsurface defects in the *Madonna* specimen applying ICT.

It should be noted that PPT, TSR, and DTT allow for simultaneous qualitative and quantitative analysis, and that there are also self-developed IRT data processing algorithms based on thermal fundamentals for the automatic segmentation and characterization of defects in different heritage objects. This is the case with the IRT works of Garrido et al. [8,29,30], focused on the surface moisture analysis in materials at different scales.

### 1.2. Deep Learning: A Brief Explanation and State of the Art in Cultural Heritage and in InfraRed Thermography

A short and condensed but clear and easy-to-read introduction to Deep Learning (DL) is presented in this section (together with the description of DL applications in cultural heritage and IRT), in order to understand every detail of the description of the DL model selected in this work (Section 2). Readers should refer to [31] for a more detailed description of DL.

DL allows the computer to perform an assigned task in an autonomous and automatic way, after a learning process of the layers of one of the DL models, by exposing these layers to the elements of the corresponding input dataset. The layers of a DL model are assimilated to a neural network where there must be more than one intermediate layer (hidden layer), apart from the input and output layer in order for the corresponding model to be considered a deep neural network (i.e., a DL model) and not a simple artificial neural network [32]. 

The assigned task to be accomplished by a DL model can be classified into one of the following strategies: supervised learning [33] or unsupervised learning [34]. If the objective task is a supervised learning, the DL model must be able to map an input data (i.e., an element of the input dataset) to an output based on example input-output pairs. In other words, the layers of the DL model learn according to examples of input–output pairs (training dataset), trying to obtain the best estimation (if the outputs are continuous values) or classification (if the outputs are discrete values) taking into account: (i) The input-output pairs of the training dataset, and (ii) the input–output pairs not used during the DL model learning process (validation dataset). In this way, the input dataset is split in two groups during the learning process (training and validation datasets), allowing the validation dataset to evaluate the DL model after its learning process on the retained input dataset in order to give more robustness to the DL model in its later use in similar input dataset and same target task (i.e., to avoid overfitting). However, in unsupervised learning, the DL model must be able to find natural groupings or structures within the input dataset (in a process known as clustering) or to reduce the input dataset dimensionality (procedure known as dimensionality reduction) without example input–output pairs [35,36]. Then, the task assigned is more specific when it is defined as supervised learning (e.g., estimating house prices from the values of surface areas, year of construction and location; or classifying images according to the animal species contained from a dataset acquired in a protected natural space), and more general when it is defined as unsupervised learning (e.g., grouping the DNA sequence of different patients in different categories in order to detect some genetic abnormality; or reducing the information contained in a point cloud for the elimination of noise and thus obtain a better 3D representation of the environment under study). It should be noted that there is a third strategy known as reinforcement learning, where the DL model focuses on finding a balance between exploration (of unexplored territory, such as unsupervised learning) and exploitation (of current knowledge, such as supervised learning) [37].

DL comes from Machine Learning (ML), which is a major subset of AI [38]. Both DL and ML use the features that describe each element of the input dataset to achieve the corresponding target task. When the number of features is close to or higher than the number of elements of the input dataset, a feature extraction process is applied to each element as an initial step in both DL and ML to prevent overfitting. Specifically, feature extraction reduces the number of features of each element of the input dataset by creating a new set of features. ML and DL then rely on the new sets of features to achieve the corresponding target tasks when the feature extraction process is applied [39]. 

The advantage of DL over traditional ML comes from the feature extraction process, in which a DL model performs the feature extraction by learning autonomously the hierarchical representation of each element of the input dataset, while a traditional ML model uses hand-crafted features. In other words, a DL model first learns by itself an implicit representation of an element of the input dataset directly along its layers, and then that model uses the learned representation to achieve the target task in that element. However, a traditional ML model employs feature extraction and uses the features obtained in a separate way, in which feature extraction represents an element of the input dataset in a valid format to achieve the target task. In addition, feature extraction in traditional ML is usually more complex and requires a more detailed knowledge of the problem domain compared to DL [40]. Therefore, DL is rather used when feature extraction is necessary (i.e., for complex problems), and traditional ML is preferred when the number of features is lower than the number of elements of the input dataset (i.e., for simple problems). Using DL in simple problems would be meaningless, since DL layers are designed to extract features first and then rely on them to achieve the target task, and would be unlikely to outperform traditional ML.

Then, the objective of the learning process of a DL model is to obtain by itself the best possible representation for each element of the input dataset, i.e., to optimize the feature extraction process. In this way, the corresponding task would be completely fulfilled without manual effort. For that, the first hidden layer of a DL model receives the features of each element of the input dataset (the elements are located in the input layer), receiving each element sequentially during one iteration (epoch) of the learning process. Then, the first hidden layer passes a new set of features (i.e., a modified version) of an element to the next hidden layer, and so on to the last hidden layer (just before the output layer). For instance, a new set of features can represent the edges of the objects contained in an image, considering as features of the image each of its original pixel values. In addition, the modified version depends on the strength of the connection (weight) between the previous and the next hidden layer. Moreover, there are as many new sets of features as there are components that form a hidden layer, where each component of a hidden layer has a specific weight with each component belonging to the previous hidden layer and adding the set of features obtained with each connection. It should be noted that the weights of a component that connect to the previous components can be different from each other and different from the weights of the remaining components belonging to the same hidden layer. Finally, the output layer groups all the sets of features obtained of an element of the input dataset along all the hidden layers (set of features ‘x’, ‘y’, ‘z’, etc., known globally as feature map) into achieving the target task [41]. 

In supervised learning, a certain estimation or classification is obtained for each element of the input dataset after one epoch of the learning process of a DL model (known as forward propagation). That estimation or classification will present an error value, modifying the weights of the components of the hidden layers of the DL model by itself (known as backward propagation) before continuing with the following epochs of the learning process to try to reduce the error presented in each element of the input dataset. It should be noted that the values of the weights related to one element are the same for the remaining elements of the input dataset [42]. Therefore, the optimum values of the weights of a DL model are those which give the minimum overall error. Typically, the overall error in the estimation or classification is often referred to with the term loss and it is calculated by the loss function. So, the closer to zero the value of the loss over the course of the epochs of the learning process is, the better weights and therefore better DL model is being obtained to fulfil the objective task. It should be noted that the loss function equation is different for each DL model used, which must represent the design goals by capturing the properties of the objective task [43].

**Figure 1:**
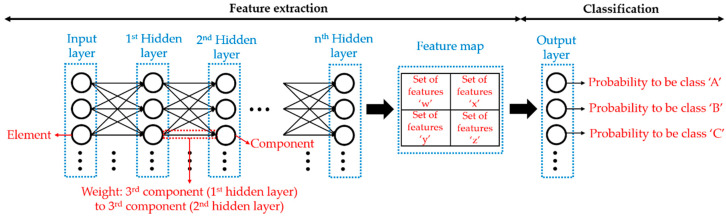
Scheme of a generic deep neural network for classification tasks indicating the terms explained in this section.

Figure 1 shows the scheme of a generic deep neural network for classification tasks.

As for the state of the art of DL, the most recent related works within cultural heritage are shown in Table 1. In IRT, each element of the input dataset can be either a thermal image or the temperature evolution of a pixel along a thermal image sequence. Regarding the features of the elements, they would be either each of the temperature values stored in the pixels of the thermal images, or each of the temperature values stored in the temperature evolutions. Table 2 shows the most recent works in IRT related to DL.

### 1.3. Motivation

Despite the good results obtained in cultural heritage with the different IRT data processing algorithms mentioned in Section 1.1.1, all of them are not automated except [8,29,30]. Automation in the interpretation of the thermal image or thermal images acquired in a case study is fundamental to minimize the operator’s subjectivity and thus maximize the accuracy of the inspections [61]. Furthermore, the possibility of online inspections through an automated interpretation is a high added value in cultural heritage monitoring. So, the development of automatic IRT data processing algorithms is a good step forward.

Therefore, this paper presents a new method consisting of the automatic detection of the defect position and its automatic segmentation (i.e., the automatic identification of the defective area), regardless of the type of defect and both surface and subsurface, from several thermal image sequences in cultural heritage. Specifically, two heritage objects with surface and subsurface defects belonging to the same family, marquetry, are analyzed with a view to serve as an initial procedure for more types of marqueteries and other heritage objects with the same and new defects in the future. 

To this end, one of the most advanced Artificial Intelligence (AI) subset currently available is introduced in the thermographic monitoring of cultural heritage, i.e., Deep Learning (DL). According to the state of the art described (Table 2), no work integrating DL and IRT for the inspection of heritage objects has been presented yet. In addition, with the purpose of further enhancing the novelty of this paper:

1. One of the latest state-of-the-art deep neural networks for object detection and segmentation tasks in images, regardless of the image spectrum, is used: Mask R-CNN [62]. According to the state-of-the-art described (Table 1 and Table 2), Mask R-CNN has neither been applied yet in cultural heritage, independently of the type of input data, nor in IRT.
2. Two automatic thermal image pre-processing algorithms based on thermal fundamentals are applied to the thermal image sequences in order to improve the contrast between defective and sound areas.

In this way, the study presents:

1. One of the most advanced DL models to automatically detect and segment different features in images. In this case, the positions and areas of defects in thermal images are determined, while reducing the limitations found with the self-developed automatic IRT data processing algorithms: (i) The low resolution of the IR cameras, (ii) the high dependence on the environmental conditions and uniformity of heating/cooling of the object under study, and (iii) the control of the different mechanisms of heat transfer [63,64].
2. A better definition of possible defects in each thermal image input to the DL model, thus increasing the performance (learning process) of the DL model.

**Figure 2:**
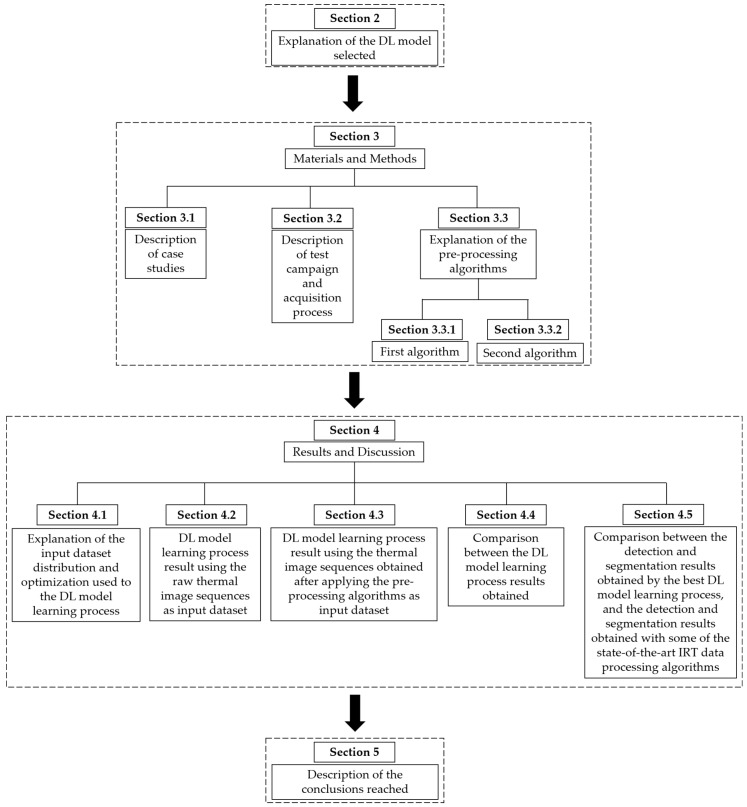
Structure followed in this work.

Figure 2 describes the structure followed in the next sections of this work.

## 2. Deep Learning Model Selected: Mask R-CNN

Mask Region-Convolution Neural Network (Mask R-CNN) is the DL model used in this work. This recent DL model developed by Facebook AI Research [62] is a simple, flexible, and general framework for object detection and segmentation tasks in images. In addition, this DL model outperforms all existing DL models in instance segmentation, bounding-box object detection, and person key point detection using the Microsoft COCO dataset [65] as input dataset. The good performance of Mask R-CNN is what led to its selection as the DL model in this work. 

It should be noted that Mask R-CNN classifies and localizes each object drawing a bounding box in the image (*object detection*) and classifies each pixel of the image into a fixed set of categories without differentiating object instances by an image mask (*semantic segmentation*). In this way, on the one hand it is possible to detect the positions of both surface and subsurface defects (*object detection*), and on the other hand it is possible to delimit their areas (*semantic segmentation*) from a thermal image in an automated way. Moreover, it is also possible to differentiate the defects classified in the same category among them by combining the two previous functions of Mask R-CNN (*object detection* + *semantic segmentation* = *instance segmentation*). 

As for the architecture, Mask R-CNN is built on top of Faster R-CNN [66], which is another DL model used in some works described in the Introduction section. Mask R-CNN consists of two stages, the first stage being identical to the first stage of the Faster R-CNN. This first stage consists of using another DL model (known as backbone DL model), which extracts a feature map from each input image (i.e., from each element of the input dataset). Then, boxes with multiple scales and aspect ratios are applied to the feature map (denoted as ‘anchor’ boxes), which serve as references to simultaneously predict object bounding boxes and object scores. Readers should refer to [66] for a better understanding of the term ’anchor’ box. The prediction process is performed by a Region Proposal Network (RPN). RPN is a kind of Fully Convolutional Network (FCN), which does not contain Fully Connected Layers (FCLs) in its architecture unlike the traditional DL models [67]. To better understand and further detail the first stage of the Mask R-CNN architecture, before explaining the second stage, convolutional, activation, pooling and upsampling layers are briefly presented and explained. 

**Figure 3:**
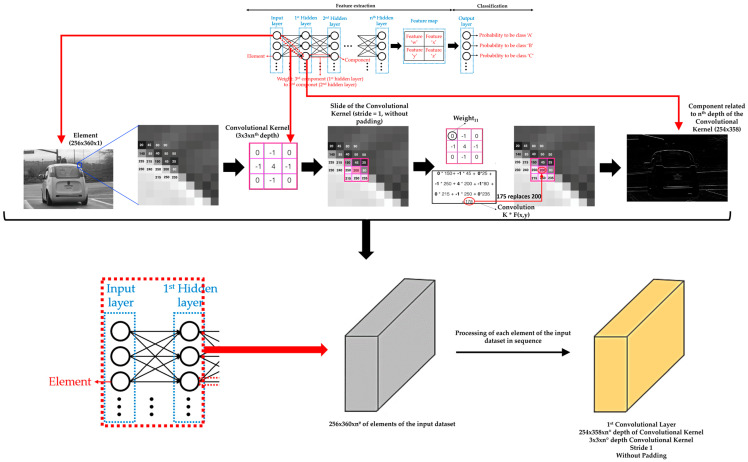
Example of a convolutional layer detailing the convolution between the first element of the input dataset and the third component of the first hidden layer. It should be noted that the dimensions of a convolutional kernel, stride, and padding between the elements of the input layer (or the components of the previous hidden layer) and the components of the next hidden layer are always the same. In addition, the dimensions of each element of the input dataset must also be the same.

A convolutional layer groups the weights between two hidden layers and the hidden layer that receives the weights. The weights of a convolutional layer are numerical values that are grouped into a tensor (convolutional kernel) of height x width x depth dimensions. Each layer of depth of a convolutional kernel determines a different importance of each part of each element of the input dataset or each part of each component of the hidden layer placed to the left of the convolutional kernel in question. In addition, a convolutional kernel has a small height and width (1 × 1 pixel, 3 × 3 pixels, 5 × 5 pixels, and so on). So, each layer of depth of a convolutional kernel slides over either the element of the input dataset or the component while performing an element-wise multiplication between its weights and the covered data of the element or component. This element-wise multiplication leads to replace the central original value of the covered data by the sum of the results of the corresponding multiplication (known as convolution). Typically, the stride of the convolutional kernel is (1,1) for the height and width movement. In short, the stride parameter dictates how big the steps are for the convolutions when sliding the convolutional kernel over either the element of the input dataset or the component [68]. Furthermore, extra rows and columns (typically, values equal to zero) are sometimes added to the edges of the element of the input dataset or the component to slide the convolutional kernel over the most external data (known as padding) [69]. As for the depth of the convolutional kernel, the number of versions (sets of features) of an element or component that passes to the next convolutional layer will depend on the depth value assigned to the convolutional kernel of that convolutional layer. In summary, a hidden layer has the same components as the depth value of the associated convolutional kernel. That is, each receptor component receives the convolution results according to the depth layer of the convolutional kernel related to each one (i.e., the weights of a component are the same for each connection with the components of the previous hidden layer), adding then in each one all the convolution results obtained. The idea is that the different depths of a convolutional kernel extract a different set of features from an element of the input dataset and that these sets of features help to achieve the assigned task. Figure 3 shows an example of a convolutional layer, adapting illustrations from reference [70].

**Figure 4:**
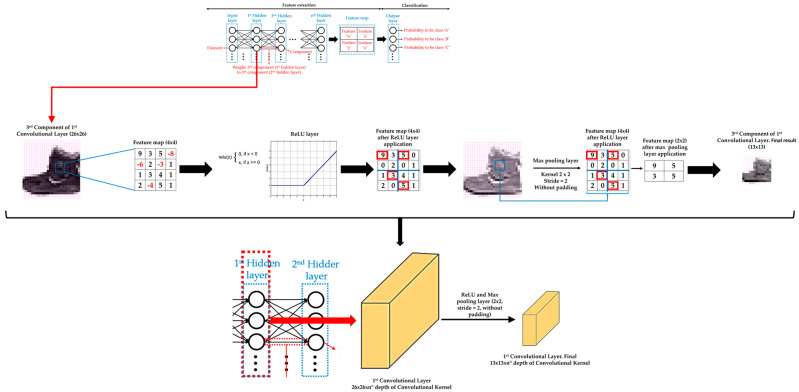
Example of an activation function layer and a pooling layer. Specifically, Rectified Linear Unit (ReLU) layer is represented as activation function layer, and max pooling layer as pooling layer.

**Figure 5:**
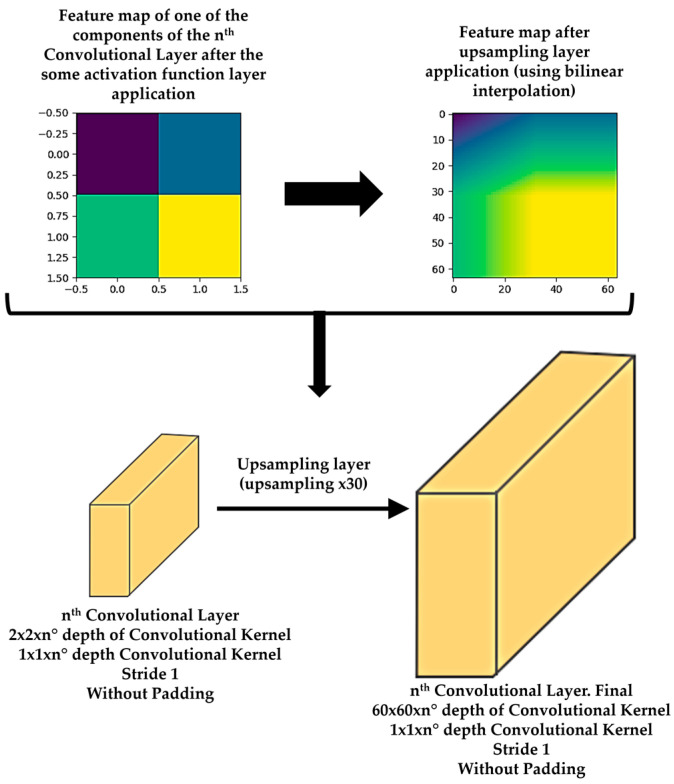
Example of an upsampling layer, using the bilinear interpolation method to upsample 30 times.

Moreover, there is usually an activation function and a pooling or upsampling layer between two consecutive convolutional layers of a DL model. An activation function layer transforms the range of values of the set of features of a component into a range that makes a DL model work better. The most commonly used activation function layer is the Rectified Linear Unit (ReLU), which converts all negative values to 0 [71]. The pooling layer downsamples each element of the input dataset or component [72], while the upsampling layer is the opposite of the pooling layer [73]. A pooling layer simplifies the information contained in the components after the application of the activation layer, summarizing the sets of features and, consequently, reducing the size of each component before moving to the next convolutional layer. Two common pooling layers are average and max pooling layer. The average and max pooling layer averages the values and selects the maximum value of the values contained in a kernel of certain dimensions (usually 2 × 2) that slides over each set of features of each component, respectively. Meanwhile, an upsampling layer works by repeating the rows and columns of the set of features of each component with some weighting (such as bilinear interpolation) before moving to the next convolutional layer. The pooling layer is required to downsample the detection of features and helps the DL model learning process to become approximately invariant to small translations of the input dataset. Meanwhile, the upsampling layer is necessary to upsample the features in order to generate an output with the same dimensions of the element of the input dataset. Then, the upsampling layer is a key layer for DL models that perform segmentation, such as Mask R-CNN. Figure 4 shows an example of an activation function layer and a pooling layer, adapting illustrations of the references [70,74], and Figure 5 shows an example of an upsampling layer, using the bilinear interpolation method to upsample, adapting illustrations from reference [75].

**Figure 6:**
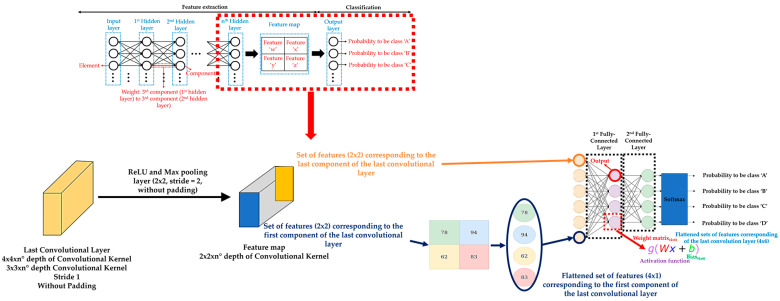
Example of Fully Connected Layers (FCLs) and a softmax activation function application.

Returning to FCLs, there is usually more than one FCL at the end of a DL model. In fact, FCL is actually the output layer of a DL model, where each set of features obtained after the pooling or upsampling layer application on the last convolutional + activation layer of the DL model is an input to the FCL. The reason is because these sets of features are based on the sets of features that have been extracted by the previous convolutional layers of the DL model. It should be noted that each set of features obtained from the last pooling/upsampling layer is flattened into a 1-D vector of length equal to the result of multiplying the height × width of the corresponding set of features before applying the FCL. Generally, the first FCL takes each flattened vector and then elementwise multiplies all the values of one of the flattened vectors with the values of the corresponding weights assigned to that flattened vector, and adds the result with the corresponding bias values. The process is repeated with all the flattened vectors. Then, an activation function (typically ReLU) is applied to each output of the first FCL before moving to the second FLC. In classification tasks, the last FCL produces a list of class scores. It should be noted that the number of outputs of the final FCL must be equal to the number of the different classes defined according to the classification task [76]. Then, a softmax activation function uses the list of class scores obtained as inputs, converting them into probabilities that sum to one, where each output of the softmax activation function is interpreted as the probability of membership for a specific class. The class with the highest probability will be the class assigned to the corresponding element of the input dataset [77]. Figure 6 shows an example of FCLs and a softmax activation function application, adapting illustrations from reference [78].

Therefore, the difference between FCNs and FCLs lies in the way they operate. The first uses convolution followed by an activation function layer, pooling layer and/or upsampling layer, and the second uses multiplication and summation followed by an activation function to each input. So, RPN obtain lists of class scores as FCLs but by changing the depth parameter of the convolutional kernels and by applying pooling layers. 

Further detailing the architecture of RPN, each ‘anchor’ box is first mapped to a lower-dimensional feature by a convolutional layer formed by a 3 × 3 × 256 convolutional kernel followed by a ReLU layer. Then, the output feature map is fed into two sibling convolutional layers: (i) One formed by a 1 × 1 × 2 convolutional kernel followed by a linear activation function, and (ii) the other formed by a 1 × 1 × 4 convolutional kernel followed by a linear activation function [79]. The first sibling convolutional layer predicts an object score to the ‘anchor’ box, and the second predicts an object bounding box to the ‘anchor’ box. The object score is the probability of an ‘anchor’ box to represent an object or not (i.e., background) after applying the softmax activation function as the last step, and the object bounding box is a refinement of the ‘anchor’ box to better fit the object after applying a regression method as the last step. Then, instead of using the softmax activation function, a regression method is applied after the second sibling convolutional layer, specifically to the 4×1 feature map obtained from each ‘anchor box’. These four features of the feature map represent the percentage change in the position (x,y) of the centroid, in the height and in the width of the corresponding ‘anchor’ box [66]. It should be noted that if there are several bounding boxes that overlap too much on the same object, the one with the highest object score (i.e., the bounding box with the highest probability of representing an object) is the only one that is not discarded after RPN by applying a technique known as Non-Maximum Suppression (NMS) [80].

Focusing on the backbone DL model, a Residual Network of 100 convolutional layers and one FCL (ResNet101)-Feature Pyramid Network (FPN) is the model selected. ResNet101 [81] is one of the most widely used DL models as feature extractor (without taking into account the FCL), of which first convolutional layers detect low-level features (e.g., edges and corners if the element of the input dataset is an image), and subsequent layers successively detect higher-level features (e.g., car, person, and sky if the element of the input dataset is an image). ResNet101 works with elements of the input dataset with 1024 × 1024 × 3 dimensions and its convolutional layers are divided into five stages: (i) First stage, a convolutional layer formed with a 7 × 7 × 64 convolutional kernel followed by a ReLU and a max pooling layer; (ii) second stage, three blocks and each having three convolutional layers formed with a 3 × 3 × 64, 3 × 3 × 64 and 3 × 3 × 256 convolutional kernel, respectively, all followed by a ReLU layer; (iii) third stage, four blocks and each having three convolutional layers formed with a 3 × 3 × 128, 3 × 3 × 128 and 3 × 3 × 512 convolutional kernel, respectively, all followed by a ReLU layer; (iv) fourth stage, 23 blocks and each having 3 convolutional layers formed with a 3 × 3 × 256, 3 × 3 × 256 and 3 × 3 × 1024 convolutional kernel, respectively, all followed by a ReLU layer; and (v) fifth stage, three blocks and each having three convolutional layers formed with a 3 × 3 × 512, 3 × 3 × 512 and 3 × 3 × 2048 convolutional kernel, respectively, all followed by a ReLU layer. It should be noted that the resulting feature map obtained after the last convolutional layer of the fifth stage has 32 × 32 × 2048 dimensions. So, the feature map size is reduced by half and the depth of the feature map is doubled at each stage of ResNet101 thanks to the max pooling layer and the strides equal to 2 in some convolutional layers [79].

As for FPN [82], this DL model is introduced as an extension of ResNet101 to better represent the features of the objects of an image at multiple scales. In this way, FPN improves the feature extractor of ResNet101. For that, FPN is a top-down architecture with lateral connections forming two different pyramids. The first pyramid takes the feature map obtained in each output of the stages of ResNet101 (except the output of stage 1). Then, each feature map is downsampled by a 1 × 1 × 256 kernel convolution, obtaining high-level features. Subsequently, the second pyramid takes the downsampled feature map of the output of the last stage of ResNet101 and upsamples it in order to add elementwise the downsampled feature map of the output of the previous stage. This last result is then upsampled again in order to add elementwise the downsampled feature map of the output of the third stage, and so on. All the outputs of the second pyramid are then subjected to a 3 × 3 × 256 convolutional kernel to create the feature maps (in total 4) used by the RPN, representing low-level features. It should be noted that in addition to applying ‘anchor’ boxes to these four feature maps, they are also applied to a fifth feature map obtained from a max pooling layer (reducing by half the dimensions) applied to the smallest feature map of the FPN.

The second stage of Mask R-CNN is different from the second stage of Faster R-CNN. In addition to applying the bounding box recognition branch to the outputs of the RPN predicted with a positive object score (RPN outputs known as candidate object bounding boxes), Mask R-CNN adds a parallel mask prediction branch. The bounding box recognition branch predicts the final object class (here the type of object is predicted as opposed to the RPN, which only differentiated between object and background), and the final object bounding box (with a better fit compared with the RPN outputs), to each candidate object bounding box. Meanwhile, the mask prediction branch outputs a binary mask for each candidate object bounding box. 

**Figure 7:**
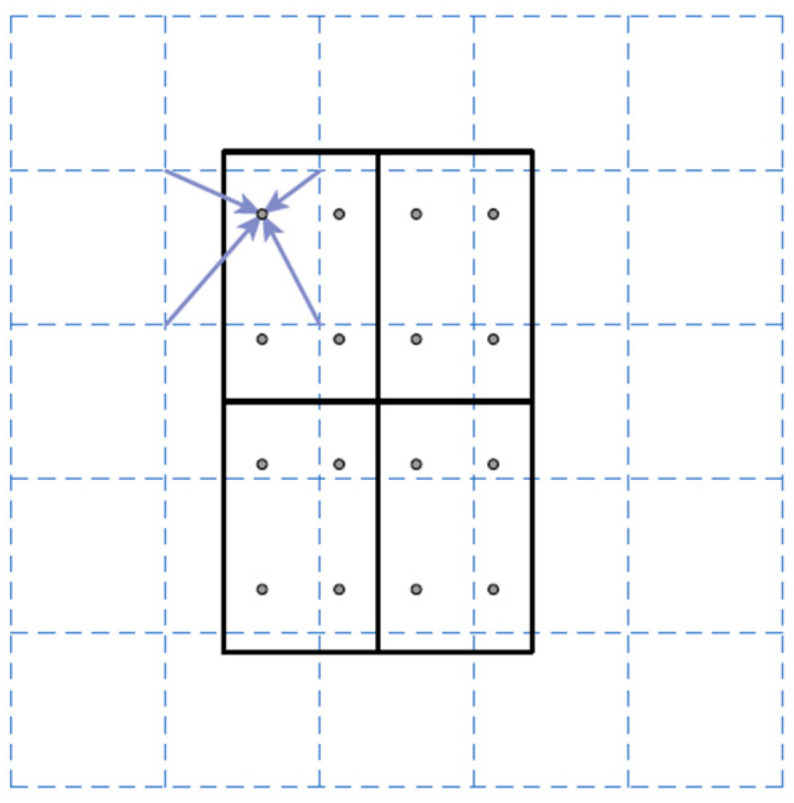
Example of a RoIAlign process (source from [83]). The dashed grid represents a depth layer of a feature map of input to Region Proposal Network (RPN) (each corner represents a different value), the solid lines represent an object bounding box divided in four parts, and the dots represent the four sampling points in each divided part. RoIAlign computes the value of each sampling point by bilinear interpolation from the nearest corners of the dashed grid (see arrows in figure).

It should be noted that a mapping operation is applied to the candidate object bounding boxes before the bounding box recognition and mask prediction branches application. A mapping operation is required in order to map the positive object scores and the object bounding boxes obtained with RPN onto the corresponding feature maps used as input to RPN. Otherwise, it would not be possible to apply the bounding box recognition and mask prediction branches with only the object scores and the values of the centroid position, width, and height of the object bounding boxes. The standard mapping operation is the Region of Interest Pool (RoIPool), which rounds down the coordinates of the four corners of each object bounding box. The boundaries of the ‘anchor’ boxes fit well into the features maps of input to the RPN. However, it is possible that the boundary of an object bounding box is dividing the values of the corresponding feature map when mapping, instead of being bounding as the ‘anchor’ boxes, due to the process of refinement. With the rounding-down process (known as quantization), the previous problem is solved, and it works well in *object detection* tasks (e.g., it is used in Faster R-CNN) [66]. However, it is not the ideal solution for *semantic segmentation*. This is because the per-value spatial correspondence of the object bounding box obtained after the RPN application is not faithfully preserved due to the rounding down process. Therefore, RoIPool is replaced by Region of Interest Align (RoIAlign) in Mask R-CNN. The main difference between RoIPool and RoIAlign is that RoIAlign does not apply the rounding down process. Instead, RoIAlign uses bilinear interpolation to compute the exact values of the feature maps in the corresponding object bounding boxes. Specifically, bilinear interpolation is applied at four regularly sampled locations places within each of the 49 equally divided parts within each object bounding box (each object bounding box is divided in 7 × 7 parts) and aggregating the different results using a max or average pooling layer. In this work, bilinear interpolation is only computed at a single point located in the center of each divided part of the object bounding box, which is nearly as effective as using four regular sample points. Figure 7 shows a RoIAlign example from [83].

Focusing on the architecture of the bounding box recognition and mask prediction branches, the first branch consists of 2 FCLs with 1024 outputs each. Since the depth of all the outputs after the FPN application is equal to 256, and each object bounding box is divided into 7 × 7 parts during the RoIAlign process, all the candidate object bounding boxes have the same dimensions (7 × 7 × 256). It should be noted that a ReLU layer is applied after each FCL, and an additional third FCL is applied, which is actually two FCLs in parallel to predict the object classes and the final object bounding boxes, respectively. In this way, the number of outputs of the first parallel FCL (FCL_31_) is equal to the number of object classes according to the assigned task, and the other FCL (FCL_32_) is equal to the multiplication of 4 (position of the centroid (x,y), weight and height of an object bounding box) by the number of object classes. Then, a softmax activation function is applied to the FCL_31_, and a regression method is applied to the FCL_32_, to obtain the final object class and the final object bounding box of candidate object bounding box, respectively [79]. 

As for the mask prediction branch, an FCN is applied to each final object bounding box of output of the bounding box recognition branch. The RoIAlign process is again applied so that the final object bounding boxes contain the corresponding feature maps used as input to RPN. In this case, each final object bounding box is divided by 14 × 14 parts during the RoIAlign process. Then, the first convolutional layer of the FCN takes as input a 14 × 14 × 256 final object bounding box, using a 3 × 3 × 256 convolutional kernel and followed by a ReLU layer. The same convolutional kernel and activation function layer is used from the second to the fourth convolutional layer. Subsequently, a transpose convolutional layer is applied, which performs an inverse convolution operation [62]. In this last layer, a 2 × 2 × 256 convolutional kernel with a stride equal to 2 and a ReLU layer is used. Thus, with that stride, the transpose convolutional layer allows doubling the dimensions of a final object bounding box (it is as a type of upsampling layer), instead of being halved with a convolutional layer. Finally, a convolutional layer with a 1 × 1 × number of object classes (according to the assigned task) convolutional kernel is applied followed by a sigmoid layer that is another type of activation function [79], obtaining a binary mask for each class and selecting as the final binary mask the binary mask containing the final object class associated with the corresponding final object bounding box coming from the bounding box recognition branch [62].

**Figure 8:**
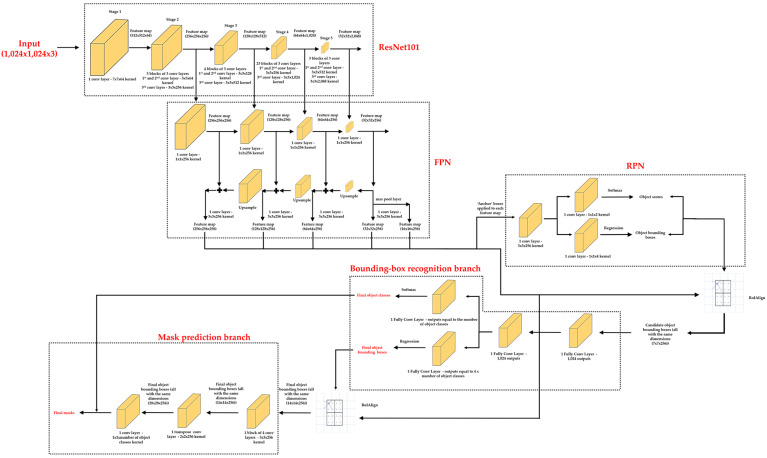
Architecture of Mask Region-Convolution Neural Network (Mask R-CNN) in its simplified version.

Figure 8 represents the architecture of Mask R-CNN in its simplified version.

Moreover, the loss function of Mask-RCNN consists of five different terms:

1. RPN_class_loss: The performance of objects can be separated from background via RPN.
2. RPN_bounding_box_loss: The performance of RPN to specify the objects.
3. MRCNN_class_loss: The performance of classifying each class of object via Mask R-CNN.
4. MRCNN_bounding_box_loss: The performance of Mask R-CNN for specifying objects.
5. MRCNN_mask_loss: The performance of the object segmentation via Mask R-CNN.

When the loss values of these five terms are smaller, the performance of Mask R-CNN improves, as indicated in the equation below.

Loss Function = RPN_class_loss + RPN_bounding_box_loss + MRCNN_class_loss + MRCNN_bounding_box_loss + MRCNN_mask_loss(1)

Readers should notice that Mask R-CNN follows supervised learning as strategy. Then, the labelling of each defect area of the marqueteries in the thermal images is performed by the VGG Image Annotator (VIA) software [84] to get the ground truth of the object classes, object bounding boxes, and object masks, and thus to compute the total loss value at each epoch of the Mask R-CNN learning process. 

## 3. Materials and Methods

### 3.1. Case Studies

**Figure 9:**
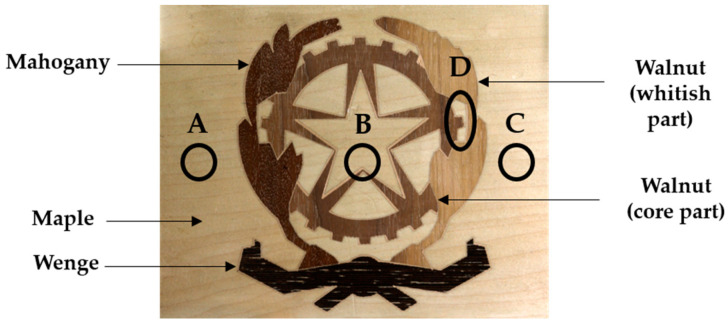
*Marquetry A* (top decorative layer) with the corresponding positions and areas of the defects.

The case studies consist of two different marqueteries. The first marquetry (hereinafter *marquetry A*) is constituted by a medium-density fiberboard of 3 mm thickness in the middle layer, and by maple wood of 0.3 and 0.6 mm thickness in the substratum and the top decorative layer, respectively. The inspected surface, i.e., the top decorative layer, also has mahogany, wenge, and walnut woods shaping the coat of arms of the Italian Republic. In addition, three artificial subsurface and one artificial surface defect have been inserted, as can be seen in Figure 9. Defect A consists of a void crossing the topmost layers of the medium-density fiberboard, while defects B and C are also voids but crossing the 3 mm thickness of the medium-density fiberboard and crossing the deepest layers of the medium-density fiberboard, respectively. As for defect D, it is a putty inserted in the top decorative layer, i.e., it is the corresponding surface defect. In this way, defects A, B, and C simulate the honeycombing effect and defect D simulates the resin (pitch) pocket effect. For detailed information on the manufacturing of this marquetry, see the work of Sfarra et al. [85].

**Figure 10:**
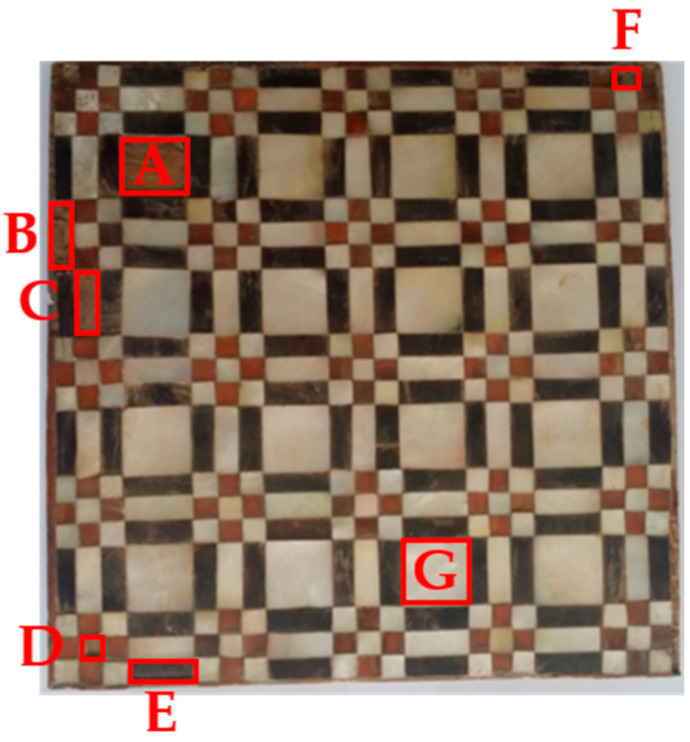
*Marquetry B* (top decorative layer) with the corresponding positions and areas of the defects.

As for the second marquetry (hereinafter *marquetry B*), it is composed of three layers as *marquetry A*. The substratum is made of fir wood, while there is an animal glue in the middle layer, and the top layer is made of multiple decorative pieces. Thus, the top layer (the inspected surface) represents a decorative layer as *marquetry A* with pieces such as pearl (white tesserae), bovine horn (some brown and black tesserae), and boxwood. Furthermore, five surface defects (A to E) and two subsurface defects (F and G) are naturally produced. The position and area of these defects can be seen in Figure 10; the surface defects represent missing tesserae, while the subsurface defects are inherent to splittings positioned at different depths.

### 3.2. Thermographic Monitoring and Acquisition Process

The use of artificial heat sources that generate a more intense thermal excitation than that generated by the solar radiation (active IRT) is necessary in order to produce a higher thermal contrast in the acquired thermal images. In this way, it is possible to identify the thermal footprints of possible subsurface defects [86,87].

Then, two FX60 BALCAR photographic lamps are used for the thermal excitation of each marquetry, which can emit heat in one of the following modes:

- Flash mode/Heat pulse. In this mode, each lamp emits 6.2 kJ of heat for 2 ms (i.e., 3.1 × 10^6^ W).
- Halogen mode/Heat wave. In this mode, each lamp emits 500 W of heat for a specific period of time (more than 2 ms).

The two heating modes are applied in each sample so that the temperature distribution and temperatures reached in the marqueteries are different both during their heating and cooling down periods. In this way, the learning process of the DL model is enriched when detecting the positions and segmenting the areas of the defects since the evolution of the temperature in them will be different with the application of either pulsed heat or waved heat. Furthermore, it is also proven that a heat pulse better identifies the thermal footprint of shallow defects, and a heat wave better identifies deeper defects in IRT [88]. 

The X6900 FLIR IR camera is the IR camera used in this work, with ResearchIR software to record the thermal images. Table 3 presents the IR camera specifications.

**Figure 11:**
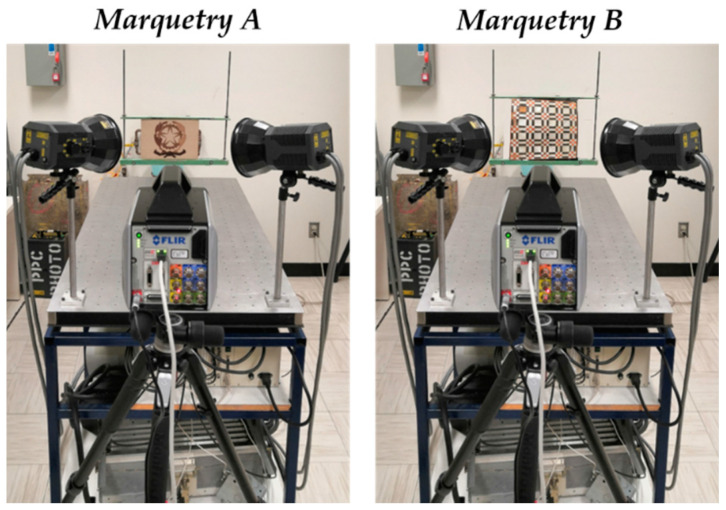
Experimental setup performed in *marquetry A* and *marquetry B*.

Moreover, Figure 11 represents the thermographic experimental setup performed, showing the relative position among the lamps, the IR camera, and the marquetry. It should be noted that the same experimental setup is used for both marqueteries.

Figure 11 shows that both the lamps and the IR camera are placed in focus on the inspected surfaces of the marqueteries (reflection mode). The lamps heat the inspected surfaces because no thermal footprint of any defect has been identified by heating the rear surfaces of the marqueteries (transmission mode). Table 4 lists the acquisition conditions that were adjusted in the experiments.

The acquisition interval has been selected as a compromise between ensuring the measurement of all the temperature steps that can arise in the marquetry and the minimum number of thermal images necessary in each experiment. The increase in the heating time in *marquetry B* with respect to *marquetry A* was due to the fact that 10 s heating highlighted no thermal footprint of any defect. The determination of the cooling time in each experiment aims to ensure the thermographic monitoring of the period of the transient cooling in each test. 

The thermal images corresponding to the transient cooling are the images of interest and the only ones used in the processing of each sequence acquired due to the following two reasons:

1. The presence of the thermal footprint of the lamps in the thermal images acquired during the heating time.
2. The quasi-invariability of the temperature over time in the thermal images corresponding to the stationary cooling (considering thermal equilibrium between the marquetry and the ambient), which are redundant images.

**Figure 12:**
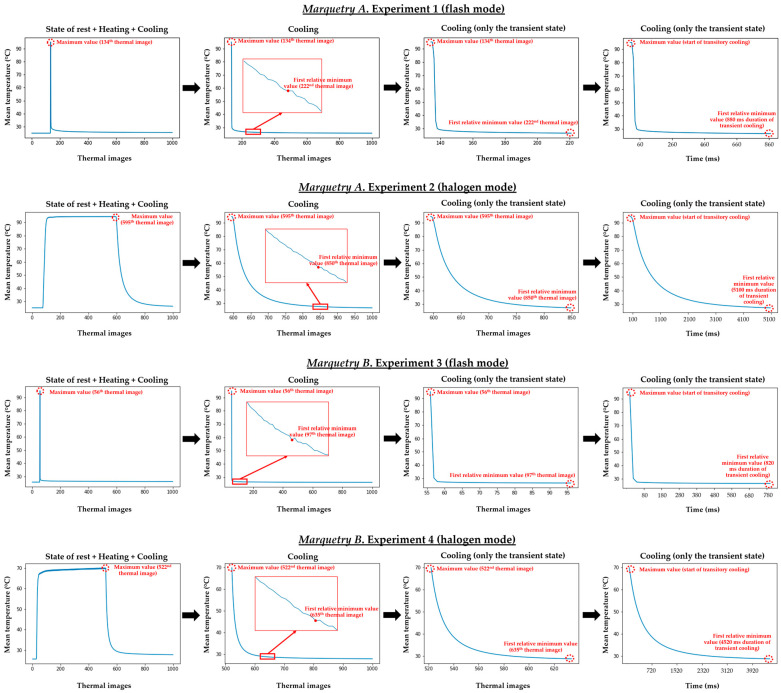
Selection of the transient cooling both in the *marquetry A* and *marquetry B* experiments.

With the purpose of selecting the period of transient cooling in each experiment, the automatic search for the maximum temperature value and the subsequent first relative minimum temperature value along the evolution of the mean temperature reached at the inspected surface is performed. Thus, the end of the heating/start of the transient cooling is defined by the maximum mean temperature, and the end of the transient cooling/start of the stationary cooling is determined with the subsequent first relative minimum mean temperature. Figure 12 shows the transient cooling obtained per test.

Since the present work is based on a qualitative analysis (defect position identification and defect area segmentation without defect depth estimation and defect - marquetry thermal characterization), neither the emissivity, nor the reflected temperature, nor the atmospheric attenuation have been compensated for in both the IR camera and the ResearchIR software. In addition, the emissivity variation and the last two parameters would also be negligible as the different parts of each inspected surface are the same or similar regarding their surface properties, and the tests are performed under laboratory conditions, respectively.

**Figure 13:**
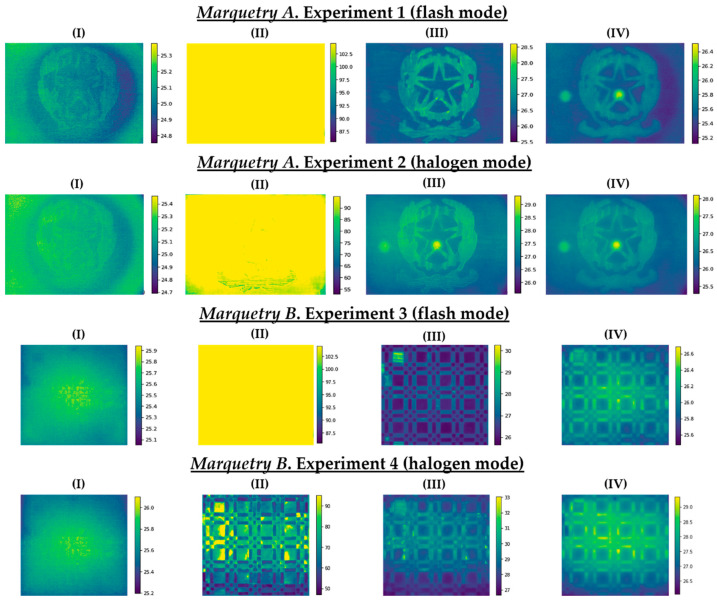
(**I**) First thermal image during the resting state at ambient conditions. (**II**) Thermal image corresponding to the end of the heating/start of the transient cooling. (**III**) Thermal image corresponding to the end of the transient cooling/start of the stationary cooling. (**IV**) Last thermal image. Temperature values are expressed in °C.

As a representation of some raw thermal images acquired, Figure 13 shows: (i) The first thermal image during the resting state at ambient conditions, (ii) the thermal image corresponding to the end of the heating/start of the transient cooling, (iii) the thermal image corresponding to the end of the transient cooling/start of the stationary cooling, and (iv) the last thermal image of each experiment (with the corresponding temperature scales in °C at the right).

No thermal footprint of the subsurface defects is appreciated in Figure 13 and only some surface defects are slightly visible with respect to the first raw thermal image of each test (column (I)). At the end of the heating/start of the transient cooling, a saturation can be seen in the thermal images of the 4 experiments, especially in the experiments where pulsed heat was applied. This is due to the fact that the temperature of the inspected surfaces has exceeded the saturation limit set in the IR camera when the thermographic monitoring was performed (column (II)). Fortunately, this phenomenon only occurs at the initial moment of the transient cooling in each test, with indications of the thermal footprint of some of the subsurface defects (especially shallower defects with flash mode and deeper defects with halogen mode) and surface defects (especially with flash mode) at the end of the transient cooling (column (III)). After the latter period, the thermal footprint of the defects is diffused due to the reach of the thermal equilibrium between the marqueteries and the environment (column (IV)). Then, with the series of thermal images corresponding to the transient cooling state of each test, a better visualization of the thermal footprint of the maximum possible number of defects is sought with the DL model used in this work, by means of the automatic identification of the position of the defects and segmentation of their areas.

### 3.3. Automatic Thermal Image Pre-Processing Algorithms

#### 3.3.1. Optimized Gaussian Model for Non-Uniform Background Heating and Cooling Compensation

This paper uses the new methodology developed by Erazo-Aux et al. [89], published in 2020. This methodology is an automatic thermal image pre-processing algorithm that compensates thermally the non-uniform background heating and cooling in thermal image sequences obtained by active IRT during the heating and cooling periods, respectively. Although the Mask R-CNN limits the high dependence of the object heating/cooling uniformity of the state-of-the-art IRT data processing algorithms, this pre-processing is proposed to improve the contrast between defective and sound areas in the thermal images of the marqueteries, thus improving the learning process of the Mask R-CNN.

This method analyses the spatial information of each thermal image from an active IRT experiment (in this case, from experiments 1 and 3 with pulsed heat, and from experiments 2 and 4 with waved heat) to automatically calculate the optimal parameters of a predefined function. This predefined function is a multivariate Gaussian distribution model, which models the spatial behavior of the non-uniform background heating or cooling by assuming that the thermal pattern on the surface of the object under study is a two-dimensional Gaussian heating or cooling distribution, regardless of the type of heat applied to the object. Equation (2) presents the multivariate Gaussian distribution model:

(2) Φk(x,θ)=A·e−12·(x−μ)T·Σ−1·(x−μ)

where Φ*^k^* is the multivariate Gaussian distribution model associated with the *k* thermal image of an active IRT experiment, *x* are the spatial coordinates of the *k* thermal image, *θ* is the vector of the unknown parameters to be calculated, *A* represents the maximum magnitude of the multivariate Gaussian distribution model (equal to (2 · *π* · |Σ|)^−1/2^), *μ* is the mean vector, and Σ is the covariance matrix. *θ* groups as unknown parameters *A*, *μ*, and Σ, calculating them automatically by using the least-squares method based on the set of temperature values of the *k* thermal image.

**Figure 14:**
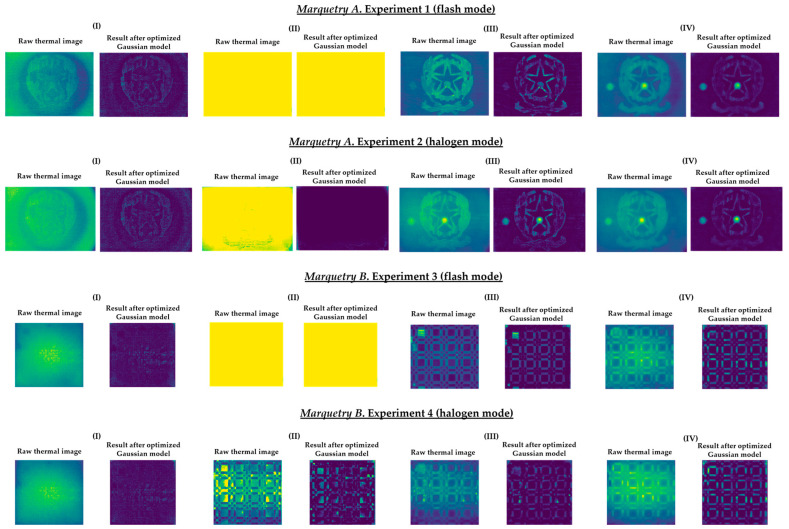
Comparison between raw thermal images and outputs after the application of the optimized Gaussian model. (**I**) First thermal image during the resting state at ambient conditions. (**II**) Thermal image corresponding to the end of the heating/start of the transient cooling. (**III**) Thermal image corresponding to the end of the transient cooling/start of the stationary cooling. (**IV**) Last thermal image.

Once the values of the unknown parameters are calculated, the estimated non-uniform background heating or cooling model for the *k* thermal image is obtained, in which the background thermal compensation results from subtracting the estimated model of the surface with non-uniform background heating or cooling in the *k* thermal image. Finally, the distribution of temperature values of the output *k* thermal image is interpolated to maintain its original width (i.e., its original range of temperature values). In this way, it is possible to compare the learning process of Mask R-CNN using as input dataset either the outputs after the application of this method or the raw thermal images. As an example, Figure 14 shows the corresponding outputs of the raw thermal images represented in Figure 13.

#### 3.3.2. Automatic Segmentation of Thermal Footprints of Possible Defects to Highlight Defect Areas Segmented

This automatic thermal image pre-processing algorithm is based on the Step 2 of the methodology proposed by Garrido et al. [8]. The algorithm consists in the search of inequalities between a thermal image just before it shows thermal footprints of defects (hereinafter, *reference_image*) and each subsequent thermal image (hereinafter, *next_image*) from a thermal image sequence. The reason for the search for these inequalities is that the temperature distribution of any defect will have its own Gaussian bell shape. Thus, analyzing the evolution of the surface through the same experiment, the shape of a thermal image histogram with thermal footprints of defects will be different from the shape of a thermal image histogram without these thermal footprints. Therefore, the segmentation of defects is performed with the search for these inequalities. Specifically, the search is automated by finding intersection points after overlapping the histograms of the *reference_image* and the corresponding *next_image*. The intersection points found in an overlapping histogram belong to ends of the Gaussian bells of the thermal footprints of defects present in the corresponding *next_image*.

The *reference_image* in this work is the thermal image corresponding to the end of the heating/start of the transient cooling in each experiment. The reason is that the possible presence of thermal footprints of defects is null or poor at that initial moment of the transient cooling. It is null or poor because the heat generated by the lamps during the heating period remains as the predominant thermal footprint. Moreover, the thermal images of the rest period are not used as *reference_image* because the thermal conditions of the marqueteries in that period are highly different from the conditions in the cooling period (the shape of the thermal image histograms between the *reference_image* and the *next_image* could be different not because of the presence of the thermal footprints of defects but because of the different distribution of the temperature in the marqueteries between being at rest and just after the application of a thermal excitation).

**Figure 15:**
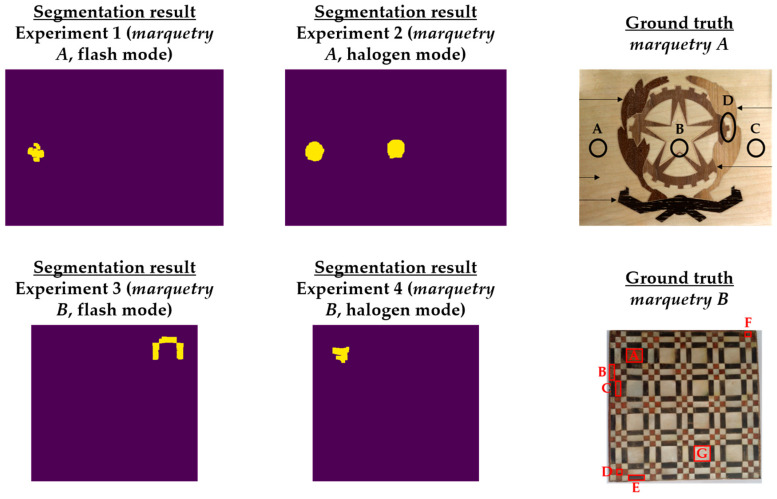
Segmentation results in each experiment after the application of the pre-processing algorithm explained in this section. The segmented areas are the yellow areas. Compared to the ground truths, the areas of defects A and B are completely segmented in experiment 2 (halogen mode) regarding the *marquetry A*, and the area of defect A is partially segmented in experiment 4 (halogen mode) regarding the *marquetry B*. Segmentation results are worse in flash mode (experiments 1 and 3), partially segmenting the defect A regarding the *marquetry A*, and erroneously segmenting regarding the *marquetry B*.

Regarding the *next_images*, as the defects of the marqueteries are static (i.e., they hardly change their areas over time, unlike the moisture propagation), one overlapping histogram is enough for each experiment. Specifically, the overlapping histograms to be applied will be those that best segment the defects, selecting the best segmentation between flash mode and halogen mode for each marquetry. Bearing this in mind, the best *next_image* is the thermal image corresponding to the end of the transient cooling, segmenting a higher extension as defects in the halogen mode than in the flash mode in both marqueteries (see Figure 15). The reasons can be the following:

1. Deeper defects take longer for their thermal footprints to become present in the thermal images (i.e., their thermal diffusions take longer to reach the surface of the marqueteries), while the thermal footprints of shallower and surface defects remain present due to the short duration of the transient cooling.
2. The segmentation of defects does not improve with the use of thermal images of the stationary cooling period, since the thermal variability of the marqueteries is quasi-nil from the end of the transient cooling period and, moreover, the thermal footprints of the defects fade with the passage of the thermal images.
3. The application of waved heat facilitates the thermal diffusion of defects more than the application of pulsed heat in both marqueteries (specially the thermal diffusion of deeper defects).

**Figure 16:**
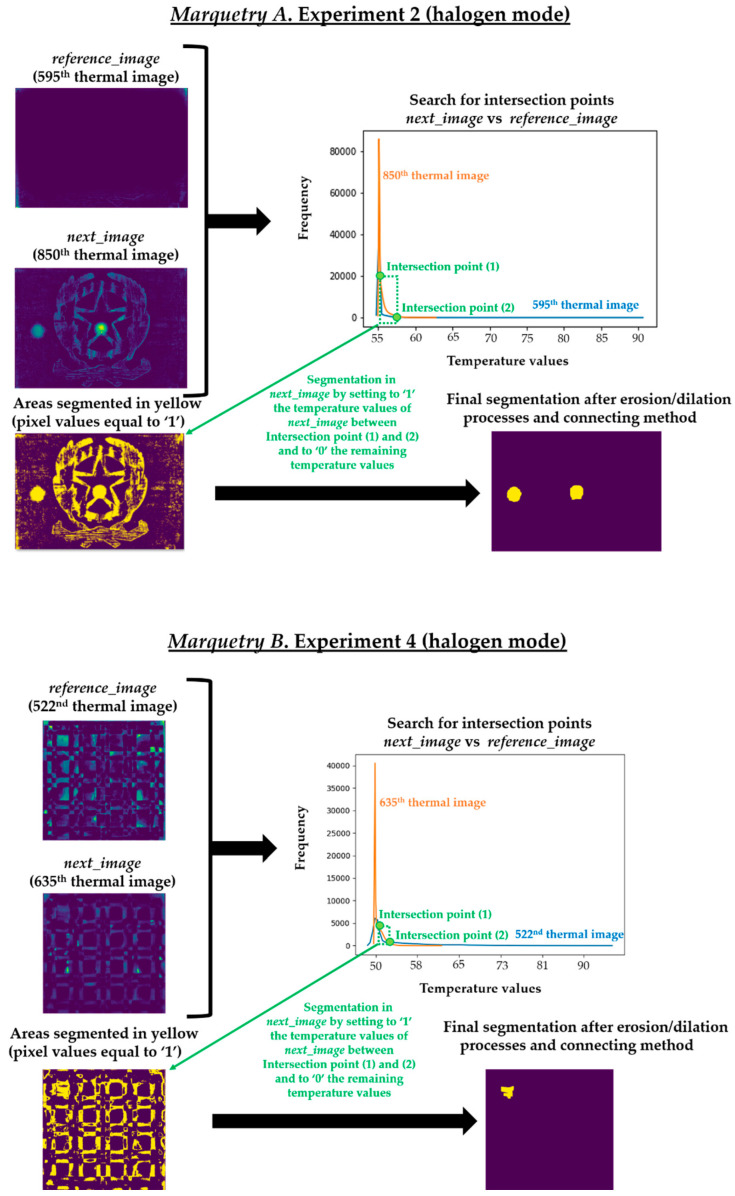
Result of each intermediate step of the segmentation process of the pre-processing algorithm proposed in this section.

It should be noted that a process of erosion and dilation [9] was applied automatically after finding the respective intersection points in each overlapping histogram and before obtaining the definitive segmenting in each *next_image* (i.e., before obtaining Figure 15). The erosion process is applied to eliminate small-size segmented areas, considered as noise, while the subsequent dilation process is applied to group the closest segmented areas into one single segmented area. In addition, each segmented area is labelled with a different index by applying a connecting method [9]. In this way, the smallest segmented areas that were not eliminated by the erosion process are automatically eliminated by counting the number of pixels in each segmented area. Figure 16 shows the result of each intermediate step of this pre-processing in the segmentation that has given the best result in each marquetry (i.e., in the experiments 2 and 4).

**Figure 17:**
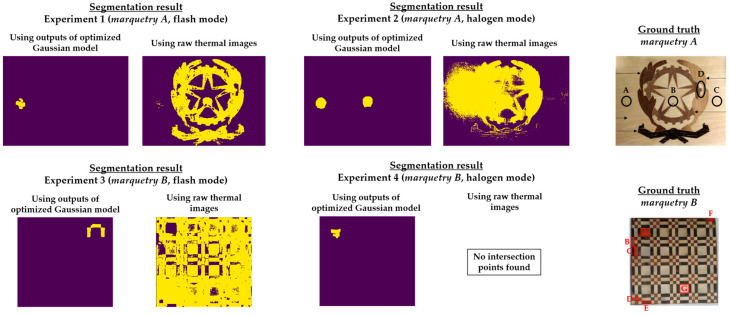
Comparison of segmentation results applying the pre-processing proposed in this section on either the outputs of the optimized Gaussian model or the raw thermal images.

It should be noted that this pre-processing is applied to the thermal image sequences obtained after the application of the optimized Gaussian model for non-uniform background heating and cooling compensation. The reason is that this proposed pre-processing would segment erroneously if applied to the raw thermal image sequences, due to the non-uniform background heating and cooling in those thermal images. As an illustration, Figure 17 shows the segmentation that would be obtained in the raw thermal image sequences using the same *reference_images* and *next_images*.

**Figure 18:**
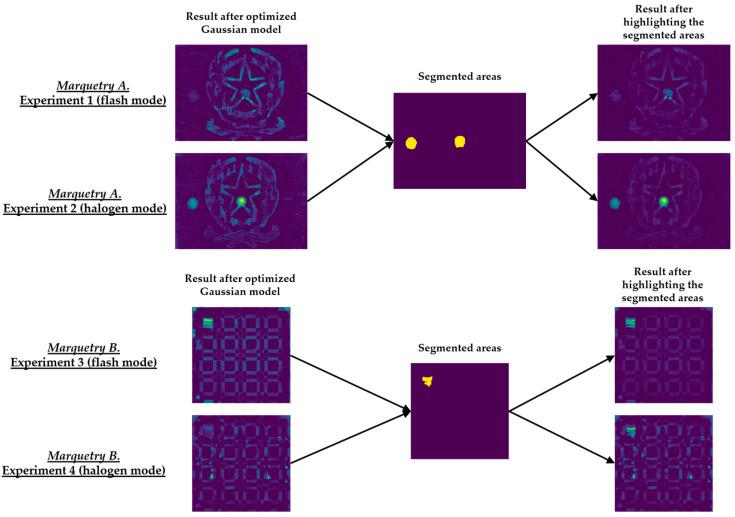
Result of the highlighting on the outputs of the optimized Gaussian model corresponding to the end of the transient cooling.

Figure 15 shows the optimal final results to be obtained with Step 2 of the methodology proposed in [8]. Figure 15 shows that it is only possible to segment the defects with the most outstanding thermal footprints (i.e., the largest and shallowest defects), although some of them can only be partially segmented. The segmentation of all the defects is not possible due to the limitations found with the self-developed automatic IRT data processing algorithms, commented in the Introduction section. Anyway, it is a useful method as pre-processing of thermal images before performing the learning process of a DL model (in this case, Mask R-CNN). It is useful because the segmentations obtained in the outputs of the optimized Gaussian model allow to highlight those segmented areas in order to increase the performance of the learning process of the Mask R-CNN. A good way of highlighting is the use of an intensity transformation operation. Specifically, the Power-Law/Gamma Transformation is used as intensity transformation operation, which modifies the histogram of the segmented areas for contrast enhancement by redistributing the density of probability. Readers should refer to [90] for more information of this intensity transformation operation. Figure 18 shows the result of the highlighting on the outputs of the optimized Gaussian model corresponding to the end of the transient cooling of each experiment. It should be remembered that the best segmentation result of each marquetry (see Figure 15) is applied to all the corresponding thermal image sequences (i.e., both the sequence obtained with the flash mode and the halogen mode). 

Section 4 compares the learning process of Mask R-CNN obtained using as input dataset: (i) The raw thermal image sequences, (ii) the thermal image sequences obtained after the optimized Gaussian model application, or (iii) the thermal image sequences obtained after the highlighting of the segmented areas on the outputs of the optimized Gaussian model, showing how the learning process is improved with (ii) and even more with (iii).

## 4. Results and Discussion

### 4.1. Input Dataset Distribution and Optimization Used for the Mask R-CNN Learning Process

Table 5 shows the distribution of the input dataset used for the learning process of Mask R-CNN. The distribution is the same for each input dataset with the purpose of making comparisons among them: (i) Raw thermal image sequences, (ii) thermal image sequences after optimized Gaussian model application, and (iii) thermal image sequences after highlighting the segmented areas on the outputs of the optimized Gaussian model.

**Figure 19:**
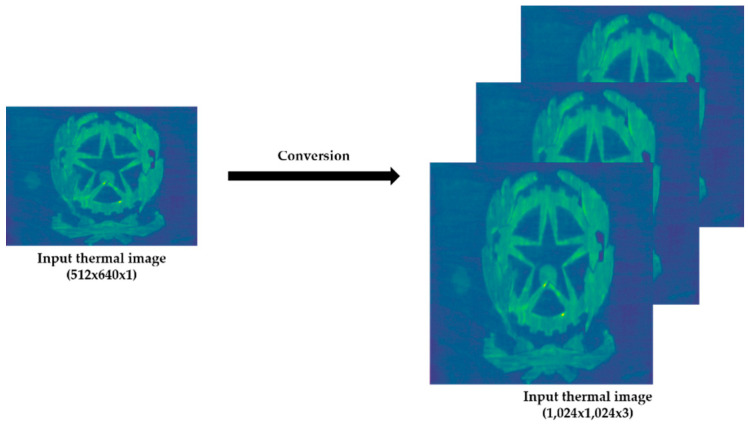
Example of an input thermal image, with the configuration required for the learning process of Mask R-CNN. It should be noted that the thermal images shown in the figures of this paper are in color in order to better appreciate the temperature value in each zone using the temperature scales shown in Figure 13 as a reference.

Moreover, each thermal image has to be resized to 1024 × 1024 and stacked two times in horizontal sequence to obtain a three-dimensional tensor, since a thermal image only has one channel. In this way, the thermal images have the correct configuration (three channels, as the case of RGB images) to be used as elements of the input dataset to the learning process of the Mask R-CNN (Figure 19).

As for the optimization used for the Mask R-CNN learning process, the hyper-parameters are set to the following values (the same values for each input dataset):

- Training_batch size = size of the training dataset.
- Validation_batch size = size of the validation dataset.
- Epochs = 100.
- Learning rate = 0.001.
- Learning momentum = 0.9.
- Weight decay = 0.1.

**Figure 20:**
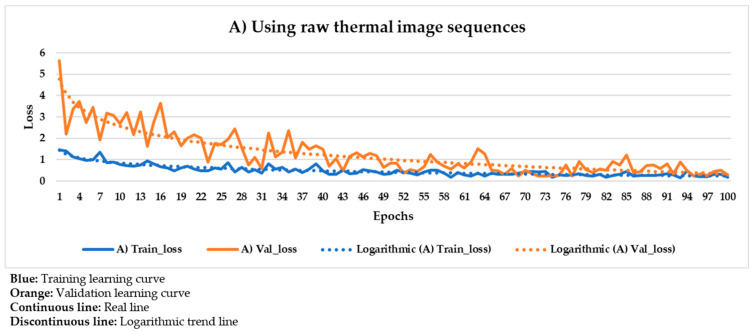
Mask R-CNN learning process result using the raw thermal image sequences as input dataset.

**Figure 21:**
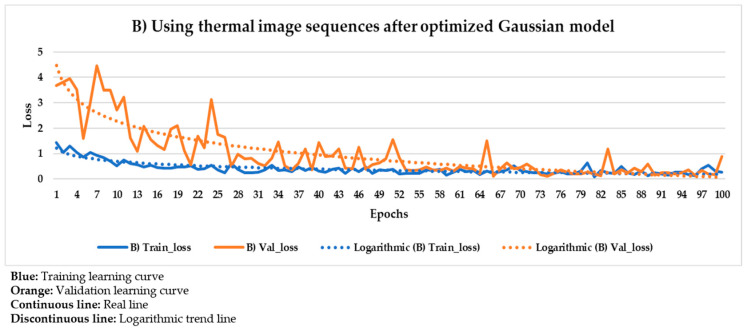
Mask R-CNN learning process result using the thermal image sequences after optimized Gaussian model as input dataset.

**Figure 22:**
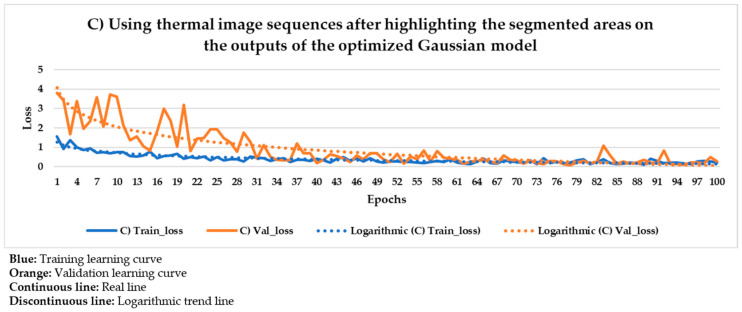
Mask R-CNN learning process result using the thermal image sequences after highlighting the segmented areas on the outputs of the optimized Gaussian model as input dataset.

**Figure 23:**
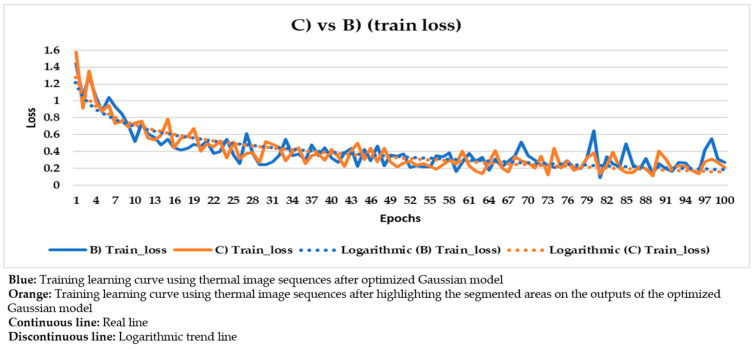
Comparison of the training learning curves obtained using the thermal image sequences after optimized Gaussian model (‘B)’) and the thermal image sequences after highlighting the segmented areas on the outputs of the optimized Gaussian model (‘C)’) as input dataset.

**Figure 24:**
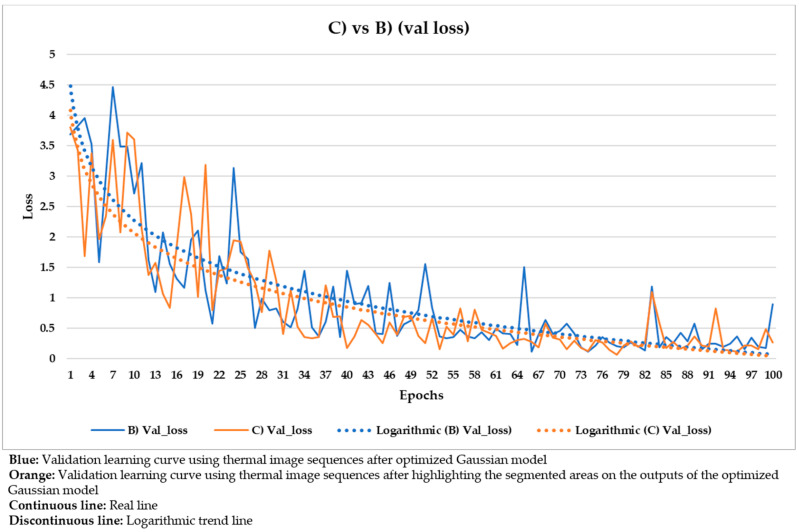
Comparison of the validation learning curves obtained using the thermal image sequences after optimized Gaussian model (‘B)’) and the thermal image sequences after highlighting the segmented areas on the outputs of the optimized Gaussian model (‘C)’) as input dataset.

**Figure 25:**
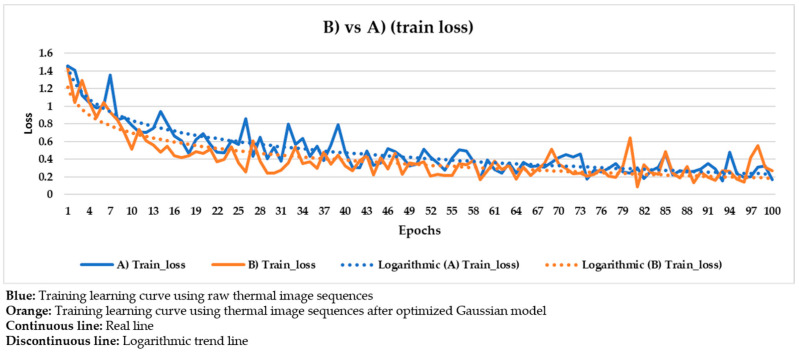
Comparison of the training learning curves obtained using the raw thermal image sequences (‘A)’) and the thermal image sequences after optimized Gaussian model (‘B)’) as input dataset.

**Figure 26:**
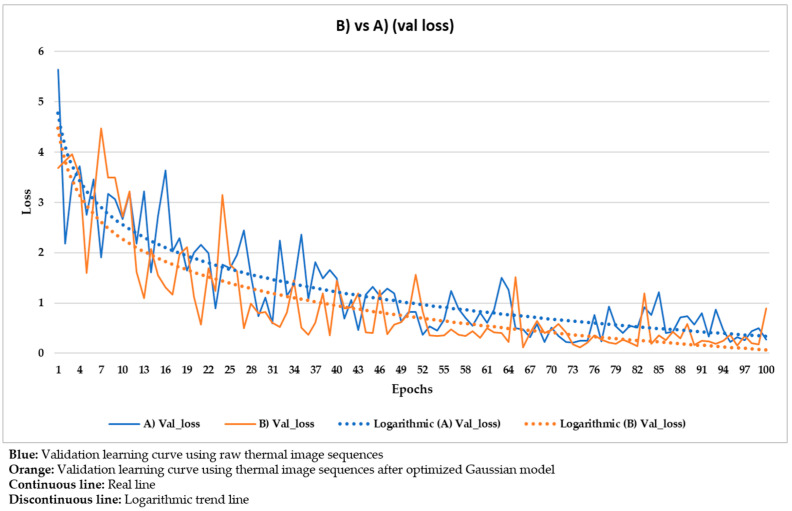
Comparison of the validation learning curves obtained using the raw thermal image sequences (‘A)’) and the thermal image sequences after optimized Gaussian model (‘B)’) as input dataset.

**Figure 27:**
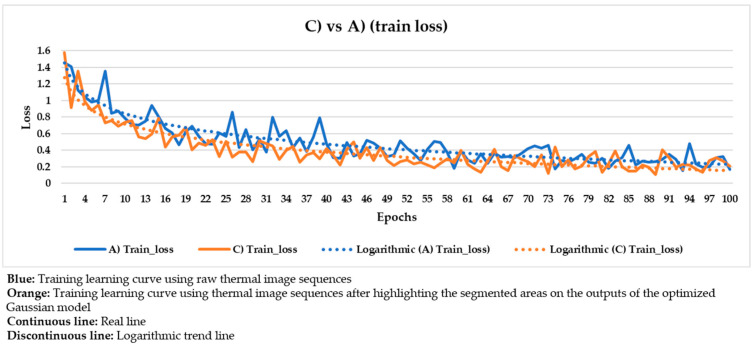
Comparison of the training learning curves obtained using the raw thermal image sequences (‘A)’) and the thermal image sequences after highlighting the segmented areas on the outputs of the optimized Gaussian model (‘C)’) as input dataset.

**Figure 28:**
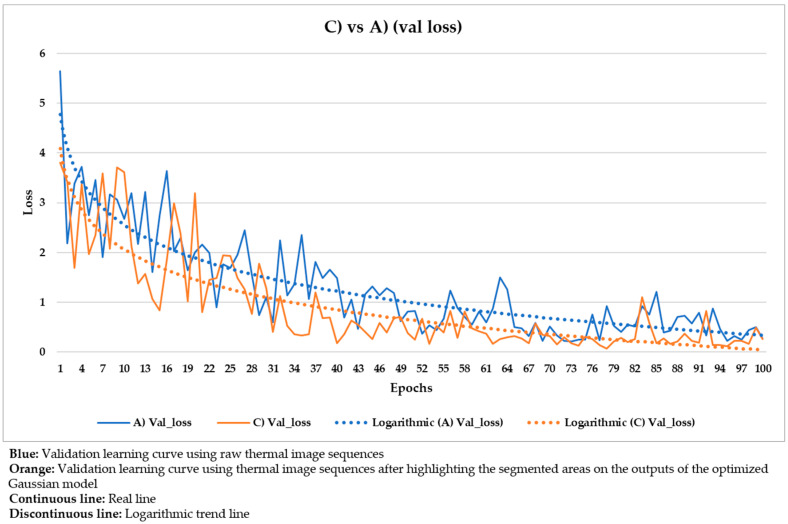
Comparison of the validation learning curves obtained using the raw thermal image sequences (‘A)’) and the thermal image sequences after highlighting the segmented areas on the outputs of the optimized Gaussian model (‘C)’) as input dataset.

These hyper-parameters are the most used and most important in the optimization of a gradient descent, the technical name of the learning process followed in Mask R-CNN. Specifically, the full name of the gradient descent is, in this case, batch gradient descent, because all the elements of the training and validation datasets are used during an epoch, and not a percentage of them randomly selected [91]. Batch gradient descent is selected because a good performance of the learning process is given more priority herein than the computation time. The number of epochs is equal to 100 because it was seen that the loss value stabilizes near the 100th epoch regarding all the different input datasets used (Figure 20, Figure 21, Figure 22, Figure 23, Figure 24, Figure 25, Figure 26, Figure 27 and Figure 28). The learning rate controls how much the DL model has to change in its weights (and also in its biases in the Mask R-CNN) in response to the estimated loss at each epoch (typical learning rate value between 0 and 1) [92]. A value of 0.001 was selected as a balance between the computational time and the search for the optimal set of weights and biases. The learning momentum smooths the progression of the learning process of a DL model and can also reduce the computational time. The typical range is between 0.9 and 0.99, with 0.9 being enough for the present work (thus avoiding an excessive smoothing) [93]. The weight decay (also known as L2 regularization method) reduces the overfitting of a DL model during its learning process, thus improving its performance. Specifically, the weight decay is a penalty for the weights of a DL model in order to avoid high changes in their values when they are updated at the end of each epoch. In other words, the weight decay limits the range of the values obtained after changes made according to the learning rate value. Reasonable values of the weight decay ranging from 0 to 0.1, with 0.1 enough herein (higher weight decay values could cause underfitting) [94].

Moreover, having a large input dataset is crucial for a good performance of the learning process of a DL model. However, only 401 and 100 thermal images are used for the training and validation datasets, respectively, and this can lead to overfitting. To increase the variety of the input datasets, some data augmentation techniques are applied. Specifically, none, some, or all of the following data augmentation techniques can be applied to the thermal images at each epoch in this work [95]:

- Horizontal flipping (63% of probability to be applied to a thermal image).
- Vertical flipping (63% of probability to be applied to a thermal image).
- Image cropping (90% of probability to be applied to a thermal image, cropping the image from 0% to 50% of its height and width).

In this way, although the size of the training and validation datasets is not increased, a certain percentage of thermal images is different between each epoch. In addition, the weights and biases obtained by Mask R-CCN after its learning process using the Microsoft COCO dataset [65] as input dataset are used herein as initial weights and biases. This is known as transfer learning, where a DL model trained for one objective task is repurposed for a second objective task [96]. In this case, the set of weights and biases of Mask R-CNN obtained after the *object detection*, *semantic segmentation*, and *instance segmentation* of 300,000 different RGB images with 80 object categories (Microsoft COCO dataset), are used as initial set of weights and biases for the *object detection*, *semantic segmentation*, and *instance segmentation* of 501 thermal images (401 to train and 100 to validate, belonging to *marquetry A* and *marquetry B*) with 1 object category (i.e., defect). The reason for using transfer learning and not an initial set of weights and biases with random values is because transfer learning accelerates the learning process of a DL model and, therefore, its performance is improved. Finally, all the layers of the Mask R-CNN are updated during the learning process (i.e., all the weights and biases are modified at each epoch), since thermal images are not natural/RGB images like the images from the Microsoft COCO dataset (having to adapt all the layers as they are grayscale images).

It should be noted that the implementation of Mask R-CNN was performed in Python, using TensorFlow and Keras as main libraries. For that, the open source code from [97] and the resources from the Supercomputing Centre of Galicia (CESGA) have been used as reference and to train and validate the proposed DL model, respectively. Specifically, the GPU employed was an NVIDIA Tesla K80.

### 4.2. 1st Mask R-CNN Learning Process: Using Raw Thermal Image Sequences as Input Dataset

Figure 20 shows the result of the Mask R-CNN learning process using the raw thermal image sequences as input dataset. 

In Figure 20, the corresponding training and validation learning curves are shown. Both learning curves converge at the end of the learning process (around the 100th epoch), being this convergence easier to visualize from the logarithmic trends than from the real evolutions. Moreover, the evolutions of the training and validation loss values stabilize in the final epochs, around 0.275 and 0.462, respectively (taking into account the loss values of the last 11 epochs, i.e., from 90th to 100th). The loss calculation is performed for the training and validation datasets separately in order to give an idea of the goodness of the DL model in the: (i) Detection of the positions of defects, (ii) segmentation of the areas of defects, and (iii) identification of different defects; in thermal images in which the weights and biases are adjusted during the learning process (training dataset) and in thermal images in which the weight and bias adjustment process is not applied (validation dataset), respectively. Thus, it is to be expected that the loss value is lower in the training learning curve than in the validation learning curve, but it is fundamental that both curves converge and have loss values close to 0 in order to consider a good performance of the learning process.

### 4.3. 2nd Mask R-CNN Learning Process: Using Thermal Image Sequences Subjected to the Pre-Processing Algorithms as Input Dataset

Figure 21 and Figure 22 show the results of the Mask R-CNN learning process using the thermal image sequences after the application of the optimized Gaussian model and the thermal image sequences after highlighting the segmented areas on the outputs of the optimized Gaussian model as input dataset, respectively.

With the purpose of better comparing between these different input datasets, the training and validation learning curves represented in the two previous figures are put together. The training and validation curves are compared in Figure 23 and Figure 24, respectively.

In Figure 21 and Figure 22, the training and validation curves converge regardless of the input dataset used. Convergence happens around the 85th and 80th epoch using the thermal image sequences after the optimized Gaussian model (input dataset ‘B)’) and the thermal image sequences after highlighting the segmented areas on the outputs of the optimized Gaussian model (input dataset ‘C)’), respectively. After both convergences, the evolutions of the training/validation losses stabilize at values around 0.275/0.322 (using input dataset ‘B)’) and 0.235/0.317 (using input dataset ‘C)’), respectively. Figure 23 and Figure 24 represent in a more direct way the improvement of the performance of the Mask R-CNN learning process by using input dataset ‘C)’ instead of input dataset ‘B)’, specifically in the lower number of epochs required for the training and validation curves convergence (5 epochs less) and in the lower training and validation loss values after the convergence (0.04/0.005 less). In addition, the logarithmic trends in Figure 23 and Figure 24 show that the training learning curve using input dataset ‘C)’ is always below the training learning curve using input dataset ‘B)’ after the 20th epoch. As for the validation learning curves, the validation learning curve using input dataset ‘C)’ is always below in all the epochs. While focusing on the real evolutions, the training and validation curves are smoother using the input dataset ‘C)’ than the input dataset ‘B)’. 

### 4.4. Comparison between the Different Mask R-CNN Learning Processes

Similar to Figure 23 and Figure 24, the following figures (Figure 25, Figure 26, Figure 27 and Figure 28) compare the training learning curves and validation learning curves between using the raw thermal image sequences (input dataset ‘A)’) and the thermal image sequences after optimized Gaussian model (input dataset ‘B)’), and between using the raw thermal image sequences (input dataset ‘A)’) and the thermal image sequences after highlighting the segmented areas on the outputs of the optimized Gaussian model (input dataset ‘C)’).

From the figures above and from the figures shown in Section 4.2 and Section 4.3, the following points are reached:

1. The performance of the Mask R-CNN learning process is better using the thermal image sequences after optimized Gaussian model than using the raw thermal image sequences, due to:
  - The lower number of epochs required for the training and validation curves convergence (from 100 to 85, 15 epochs less).
  - The lower training and validation loss values after the convergence using the thermal image sequences after optimized Gaussian model, compared with the last 11 epochs using the raw thermal image sequences (from 0.275/0.462 to 0.275/0.322, 0/0.14 less).
  - The training and validation learning curves using the thermal image sequences after optimized Gaussian model are always below in all the epochs (observing the logarithmic trends).
  - The training and validation learning curves are smoother using the thermal image sequences after optimized Gaussian model (observing the real evolutions).
2. The performance of the Mask R-CNN learning process is even better using the thermal image sequences after highlighting the segmented areas on the outputs of the optimized Gaussian model (see the improvement in Section 4.3) than using the raw thermal image sequences, due to:
  - The lower number of epochs required for the convergence of the training and validation curves (from 100 to 80, 20 epochs less).
  - The lower training and validation loss values after the convergence using the thermal image sequences after highlighting the segmented areas on the outputs of the optimized Gaussian model, compared with the last 11 epochs using the raw thermal image sequences (from 0.275/0.462 to 0.235/0.317, 0.04/0.145 less).
  - The training and validation learning curves using the thermal image sequences after highlighting the segmented areas on the outputs of the optimized Gaussian model are always below in all the epochs (observing the logarithmic trends).
  - The training and validation learning curves are smoother using the thermal image sequences after highlighting the segmented areas on the outputs of the optimized Gaussian model (observing the real evolutions).

### 4.5. Comparison between the Detection and Segmentation Results Obtained by the Best Mask R-CNN Learning Process and by Some of the State-of-the-Art IRT Data Processing Algorithms

**Figure 29:**
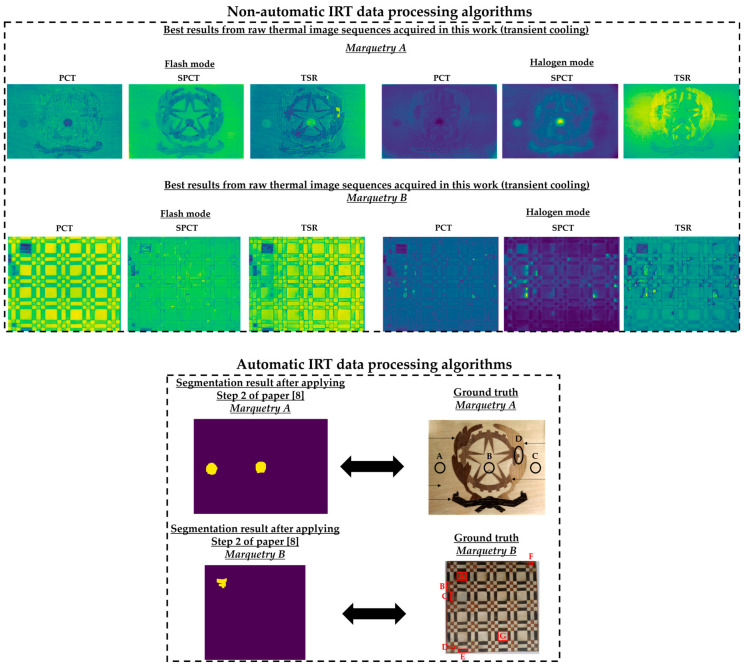
Results obtained with some state-of-the-art InfraRed Thermography (IRT) data processing algorithms applied to the marqueteries under inspection.

**Figure 30:**
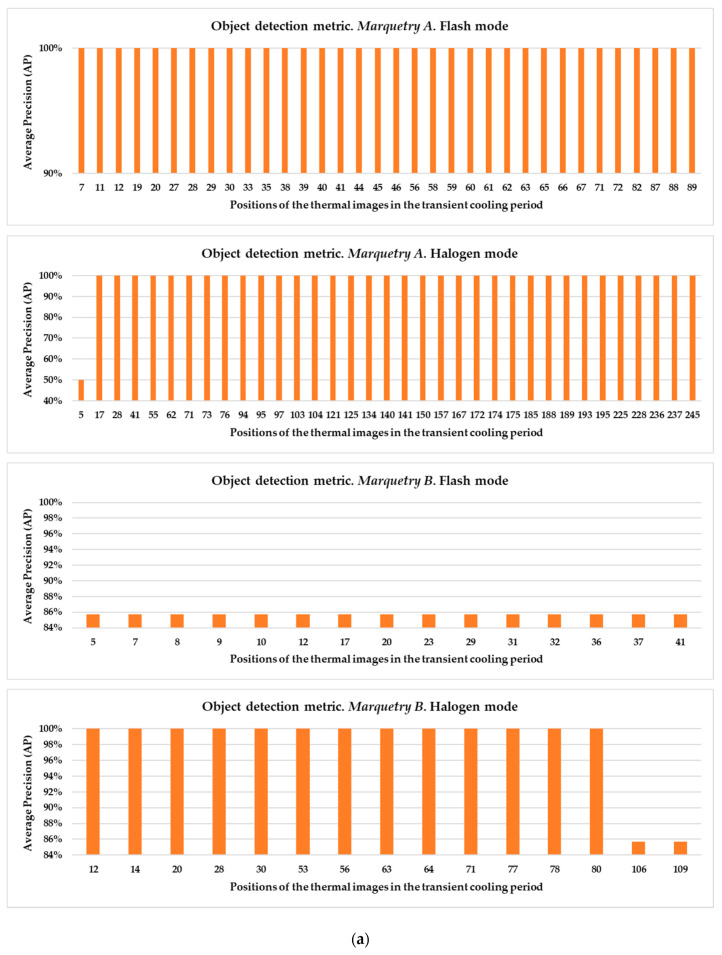

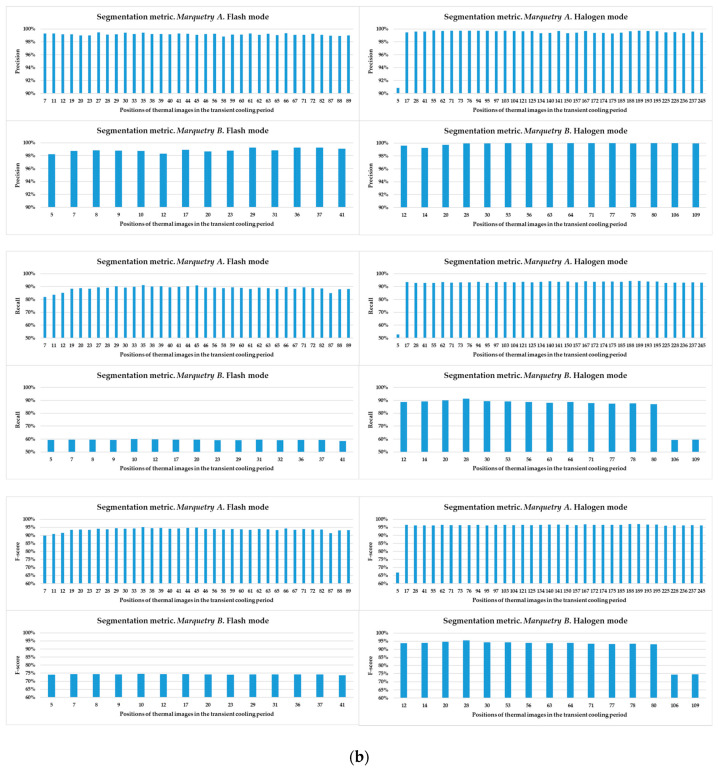

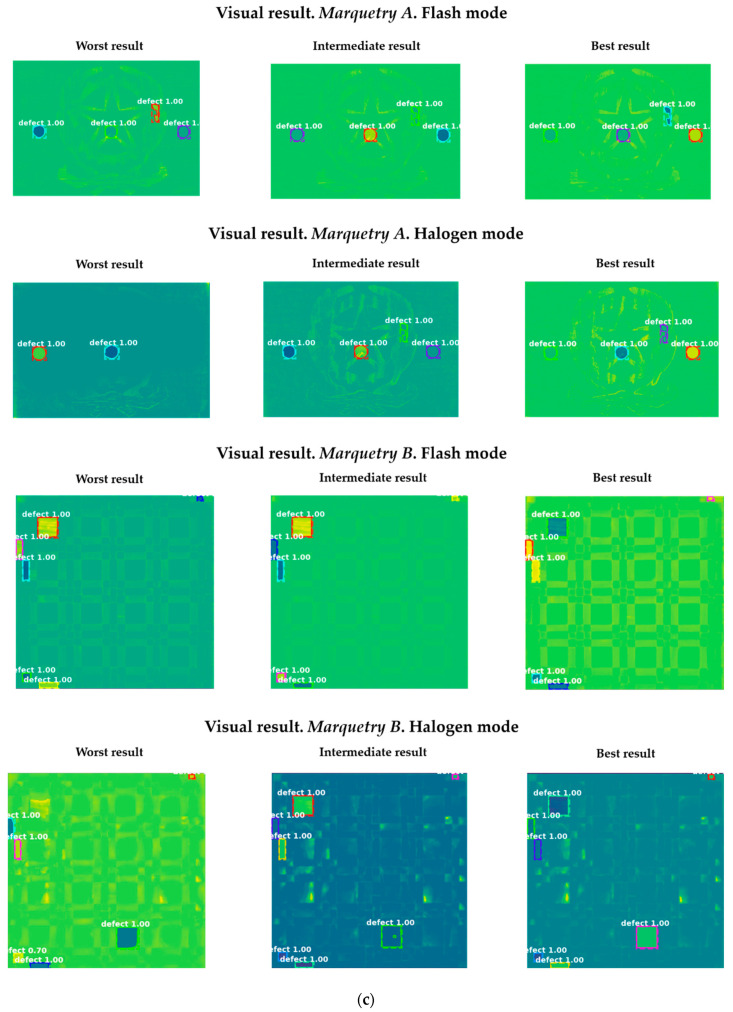
(**a**) Defect position detection metric applying the set of weights and biases obtained in the best Mask R-CNN learning process in the validation dataset. (**b**) Defect area segmentation metric applying the set of weights and biases obtained in the best Mask R-CNN learning process in the validation dataset. (**c**) Visual result of the worst, intermediate and best defect position detection and defect area segmentation applying the set of weights and biases obtained in the best Mask R-CNN learning process in the validation dataset.

This section shows how the results of the position detection and area segmentation of each defect existing in the two marqueteries are better: (i) With the latest state-of-the-art DL model for object detection and segmentation tasks in images (Mask R-CNN), using the results of its best learning process (i.e., using the thermal image sequences after the application of the two thermal image pre-processing algorithms presented herein as input dataset), than (ii) using state-of-the-art IRT data processing algorithms. Figure 29 shows the results that would be obtained with some state-of-the-art IRT data processing algorithms, while Figure 30a–c) shows the corresponding detection and segmentation results of the Mask R-CNN using the set of weights and biases obtained at the end of the best learning process in the validation dataset. It should be noted that another set of weights and biases could be used from the convergence of the training and validation learning curves (i.e., from 80th epoch), since the training and validation loss values are stabilized. This set of weights and biases is applied in the validation dataset to see the robustness of the DL model in thermal images that have not been used during the weight and bias adjustment process.

The Average Precision (AP) is a popular metric in measuring the accuracy of the object bounding boxes predicted in each element of the input dataset. For that, this object detection metric computes the average precision value for recall value over 0 to 1. Readers should refer to [98] for more information. The precision and recall parameters are also used to measure the segmentation performance of a DL model, together with the F-score parameter. The corresponding equations are as follows:

Precision = *TP*/(*TP* + *FP*) (3)

Recall = *TP*/(*TP* + *FN*) (4)

F-score = 2(Precision·Recall)/(Precision + Recall)(5)

where *TP*, *FP*, and *FN* are the True Positives, False Positives, and False Negatives of the segmentation results, compared with the corresponding ground truths, respectively. It should be noted that the reason for using F-score is that this performance metric takes into account both the precision and recall parameters, thus being the most representative.

Comparing Figure 29 and Figure 30, the deepest defects are not identified with the state-of-the-art IRT data processing algorithms (defect C in *marquetry A*, and defects G and F in *marquetry B*). However, with the methodology proposed in this work, the detection of the positions of the defects (Table 6) and the segmentation of the areas of the defects (Table 7) in the marqueteries are successful and automated as an added value. The worst detection and segmentation results are generally obtained at the beginning of the transient period in each experiment, since the thermal footprints of the defects are not very noticeable yet, although in the *marquetry B* in halogen mode, the worst results are towards the end of the transient period probably because there would begin to be signs of the diffusion of the thermal footprints of the defects. Finally, all the detections and segmentations made were predicted with a 100% probability of defect (and then 0% probability of background).

## 5. Conclusions

This work introduces DL in the thermographic monitoring of cultural heritage for the automatic detection of the defect positions and the automatic segmentation of the defect areas, regardless of the defect type and defect depth. For that, two different types of marquetry have been used as heritage elements, the first with one surface defect (simulating the resin pocket effect) and three subsurface defects (simulating the honeycombing effect), and the second with five surface defects (representing different missing tesserae) and two subsurface defects (simulating detachments). As for the monitoring, two different experiments have been applied to each marquetry, one heating the marqueteries with pulsed heat (*pulsed thermography*) and the other heating the marqueteries with waved heat (*step-heating thermography*). The thermal images belonging to the transient cooling period have been selected as the thermal images of interest in each experiment due to the higher presence of the thermal footprints of the defects than in the thermal images belonging to the heating period and the stationary cooling period.

As an added value, the latest state-of-the-art DL model for object detection and segmentation tasks in images has been selected in this work: Mask R-CNN. This DL model makes it possible to detect the position of the different defects by bounding boxes (*object detection*) and to segment the areas of the defects by binary masks (*semantic segmentation*), with a certain probability that they are really defects and not background. By combining the bounding boxes and the binary masks obtained, the differentiation between the different defects as different instances is also achieved (*instance segmentation*). Moreover, in addition to the typical optimization methods used to improve the performance of a DL model during its learning process (appropriate values of the hyper-parameters, data augmentation, and transfer learning), two automatic thermal image pre-processing algorithms based on thermal fundamentals have also been applied to the thermal image sequences used for the learning process (input dataset) of the Mask R-CNN. Both thermal image pre-processing algorithms improve the contrast between defective and sound areas, the first one by compensating the non-uniform background heating and cooling, and the second one by highlighting the segmented areas obtained in the outputs of the previous pre-processing algorithm that represent the total or partial area of the defects with the most outstanding thermal footprints. The purpose is to demonstrate how it is possible to improve the performance of DL models applied to thermographic data by combining them with thermal fundamentals.

The results obtained from the learning process of Mask R-CNN were promising in two aspects:

1. In being able to automate the interpretation of the acquired thermal images with a high percentage of success in the detection and segmentation of defects. With the state-of-the-art IRT data processing algorithms, the identification of the deepest defects of the marqueteries is not possible, neither with non-automatic algorithms (such as PCT, SPCT, and TSR) nor with self-developed automatic algorithms (without using DL).
2. In the reduction: (i) In time (epochs), (ii) in *object detection*, *semantic segmentation*, and *instance segmentation* errors (loss), (iii) and in learning instability (learning curve); using the resulting thermal images after the application of the proposed pre-processing thermal image algorithms instead of using the corresponding raw thermal images.

In summary, this work takes the first step in the use of DL models for the inspection of cultural heritage with thermographic data, using one of the best DL models currently available and even improving its performance by using algorithms that exploit the thermal information contained in the thermal images. The robustness of the DL model trained in this paper will probably be acceptable when applied to other types of marqueteries and other heritage objects with the same and/or different defects. The reason for this is that: (i) Two different experiments have been performed on each marquetry (*pulsed thermography* and *step-heating thermography*), (ii) both marqueteries have materials commonly used on decorative surfaces of heritage objects, and (iii) several types of defects with different sizes are located at different positions and depths. In any case, future research will continue with the joint application of DL and IRT data pre-processing algorithms in thermographic monitoring of cultural heritage. Especially, future research will be based on the analysis of the same and/or other defects in more types of marqueteries and other artistic objects in order to: (i) Classify between different types of defects (and not only classify between defect and background), and (ii) train a DL model with a higher variety of defects and objects towards a more robust learning. Finally, the automatic estimation of the defect depth is another point to be considered.

## Figures and Tables

**Table 1:** Comparative table of the most recent Deep Learning (DL) works within cultural heritage.

Work [Ref.]	Main Objective	Input Dataset	DL Model	Result
Dung and Anh [44]	Development of a superficial crack detection and segmentation method	Concrete crack dataset of 40,000 RGB images of 227 × 227 pixels	KittiSeg network	Overall classification accuracy of at least 97.8%
Cha et al. [45]			Self-developed DL model	Overall classification accuracy of approximately 98%
Gibb et al. [46]		Concrete crack dataset of RGB images	Self-developed DL model, using a Genetic Algorithm (GA) to optimize the following parameters of a DL model that control the structure of the deep neural network during its learning process: network depth, layer size and hyper-parameters	Overall classification accuracy of up to 89.17%
Hatir et al. [47]	Development of a weathering detection and segmentation method	8598 RGB images of historical stone monuments which contain fresh rock and eight different weathering types (flaking, contour scaling, cracking, differential erosion, black crust, efflorescence, higher plants, and graffiti)	Self-developed DL model	Overall classification accuracy of 99.4%
Llamas et al. [48]	Development of a classification method of cultural architectural heritage	More than 10,000 RGB images and 10 categories of elements defined (altar, apse, bell tower, column, dome inner, dome outer, flying buttress, gargoyle (and chimera), stained glass and vault)	Several DL models (Alexnet, Inception V3, ResNet and Inception-ResNet-V2)	More than 90% of overall classification accuracy with the best DL model
Pierdicca et al. [49]		11 points clouds (2,613,248 points in total) representing indoor and outdoor scenes of churches, chapels, cloisters, porticoes, and loggias. 10 categories of elements defined (arc, column, door, floor, roof, stairs, vault, wall, window, and decoration)	Dynamic Graph Convolutional Neural Network (DGCNN), modifying the input layer to add other meaningful elements for point cloud in addition to the X, Y and Z coordinates of each point, such as the normal value and color intensity	Overall classification accuracy about 80%

**Table 2:** Comparative table of the most recent DL works within IRT.

Work [Ref.]	Main Objective	Input Dataset	DL Model	Result
Hu et al. [50]	Development of a superficial crack detection method	Several thermal image sequences belonging to different metal samples, including ferromagnetic materials with artificial cracks, non-ferromagnetic materials with artificial cracks and non-ferromagnetic materials with natural cracks	Faster Region-Convolution Neural Network (Faster R-CNN)	Overall probability of detection of 97%
Duan et al. [51]	Development of a foreign matter invasion detection and segmentation method	(1) Several thermal image sequences belonging to two stainless steel plates with flat-bottomed-holes that simulate foreign matter invasions (air, oil and water), and (2) the corresponding coefficients obtained after the TSR application	Self-developed DL model	Overall classification accuracy of over 90% using the coefficients obtained after the TSR application instead of the thermal images as input dataset
Ali [52]	Development of a subsurface damage detection method	34 thermal image sequences belonging to different steel elements of the century-old Arlington Bridge in Winnipeg, Canada	Deep Inception Neural Network (DINN)	Maximum overall classification accuracy of 96%
Yousefi et al. [53]	Development of a subsurface defect detection method	Several thermal image sequences belonging to a steel sample and a Carbon Fiber Reinforced Polymer (CFRP) composite plate. The steel specimen has 7 bottom holes of various sizes (4 mm to 30 mm) and depths (3 mm to 9 mm), and the CFRP plate has several handmade defects created at different depth ranges, 0.2 mm to 1 mm	ImageNet-VGG-f	Promising performance for the application of heating and cooling based active IRT with a reasonable computational cost
Bang et al. [54]	Development of a superficial defect detection method	Several thermal images belonging to different composite materials	Faster R-CNN	Maximum overall classification accuracy of 75%
Luo et al. [55]	Development of a subsurface debond defect detection and segmentation method	(1) Several thermal image sequences to the spatial DL model, and (2) the temperature evolution at each pixel to the temporal DL model, from the thermal image sequences used in (1). Thermal data belong to regular and irregular shape CFRP specimens.	VGG-Unet (spatial DL model) and 3-layer-LSTM (temporal DL model)	Overall probability of detection of 66.7% with the spatial DL model, and probability of detection of up to 100% with the temporal DL model, although in some samples it was 0%
Fang et al. [56]	Development of a subsurface defect detection method	Several thermal image sequences belonging to materials of different types: CFRP, Glass Fiber-Reinforced Polymer (GFRP), Plexiglas (Plexi) and steel	Yolo-v3	Maximum overall classification accuracy of 99.8%
Cheng et al. [57]	Development of a concrete delamination detection and segmentation method	Several thermal image sequences belonging to a bridge deck	Customized DL model based on the encoder-decoder architecture (using DenseNet and DenseASPP on the encoder side)	Maximum overall classification accuracy of 51%
Habaibeh et al. [58]	Development of a method for predicting future savings in an existing building after a retrofitting	Thermal images and historical weather data related to an existing building, which insulation and solar photovoltaic panels are introduced	Self-developed DL model	Heat loss prediction with an accuracy of over 82%
Janssens et al. [59]	Development of a method for determining the condition of machines	Several thermal image sequences related to the operation of a rotatory machine	VGG	Accuracy of 95% and 91.67% for machine-fault detection and oil-level prediction
Ornek and Ceylan [60]	Development of a healthy and unhealthy neonate classification method	3800 raw thermal images and 11,400 augmented thermal images belonging to 19 healthy and 19 unhealthy neonates	Self-developed DL model	Overall classification accuracy over 99% (with a 26.29% increase due to the use of the augmented thermal images)

**Table 3:** Specifications of the InfraRed (IR) camera used.

Model	X6900 FLIR
Sensor	InSb CCD matrix
NETD	<40 mK
Thermal image/pixels	640 (H) × 512 (V)
Intensity resolution	14 bits
Accuracy	±1 °C or ±1% of reading, whichever is greater
Spectral range (μm)	3 to 5

**Table 4:** Acquisition conditions for the experiments performed.

Experiment	1	2	3	4
Marquetry	*Marquetry A*		*Marquetry B*	
Heat generated	Pulsed heat	Waved heat	Pulsed heat	Waved heat
Ambient conditions	Laboratory (ambient temperature = 22.5 °C, relative humidity = 30%)			
Camera-to-marquetry distance/angle with regard to the perpendicular of the marquetry	73 cm/0°			
Lamps-to-marquetry distance/angle with regard to the perpendicular of the marquetry	45 cm/25°			
State of rest/Heating time/Cooling time	1.33 s/2 ms/8.667 s	1.52 s/10 s/8.48 s	1.1 s/2 ms/18.898 s	1.12 s/20 s/18.88 s
Sampling rate/Number of thermal images acquired	100 Hz/1000	50 Hz/1000	50 Hz/1000	25 Hz/1000

**Table 5:** Input dataset distribution for the Mask R-CNN learning process. The selection of the thermal images of the transient cooling in each experiment for the training and validation datasets is random.

Experiment	Marquetry	Number of Thermal Images for the Training Dataset (% of the Total Number)			Number of Thermal Images for the Validation Dataset (% of the Total Number)			Training/Validation
1	*Marquetry A*	54 (13.5%)	275 (68.6%)	401 (100%)	35 (35%)	70 (70%)	100 (100%)	401 (80%)/100 (20%)
2		221 (55.1%)			35 (35%)			
3	*Marquetry B*	27 (6.7%)	126 (31.4%)		15 (15%)	30 (30%)		
4		99 (24.7%)			15 (15%)			

**Table 6:** Detection results obtained.

	*Marquetry A*		*Marquetry B*	
	Flash mode	Halogen mode	Flash mode	Halogen mode
AP	100% (except in one thermal image in halogen mode with 50%)		Near 86%	100% (except in two thermal images near 86%)
mean AP	97.07%			

**Table 7:** Segmentation results obtained.

	*Marquetry A*		*Marquetry B*	
	Flash mode	Halogen mode	Flash mode	Halogen mode
Precision	Between 98% and 100% (except in one thermal image in *marquetry A* in halogen mode with near 91%)			
mean Precision	99.28%			
Recall	Between 80% and 90%	Near 93% (except in one thermal image near 53%)	Near 60%	Near 90% (except in two thermal images near 60%)
mean Recall	84.95%			
F-score	Between 90% and 97% (except in one thermal image with near 90% and near 67.5% in flash and halogen mode, respectively)		Near 75%	Near 95% (except in two thermal images near 70%)
mean F-score	91.04%			

## Data Availability

No new data were created or analyzed in this study. Data sharing is not applicable to this article.

## References

[1] Blake J. (2000). On Defining the Cultural Heritage. Int. Comp. Law Q..

[2] Bowitz E., Ibenholt K. (2009). Economic impacts of cultural heritage-Research and perspectives. J. Cult. Herit..

[3] Yilmaz H.M., Yakar M., Gulec S.A., Dulgerler O.N. (2007). Importance of digital close-range photogrammetry in documentation of cultural heritage. J. Cult. Herit..

[4] Maldague X.P. (2001). Theory and Practice of Infrared Technology for Nondestructive Testing.

[5] Garrido I., Lagüela S., Arias P. (2018). Infrared Thermography’s Application to Infrastructure Inspections. Infrastructures.

[6] Tavakolian P., Sfarra S., Gargiulo G., Sivagurunathan K., Mandelis A. (2018). Photothermal coherence tomography for 3-D visualization and structural non-destructive imaging of a wood inlay. Infrared Phys. Technol..

[7] Chulkov A.O., Sfarra S., Saeed N., Peeters J., Ibarra-Castanedo C., Gargiulo G., Steenackers G., Maldague X.P.V., Omar M.A., Vavilov V. (2020). Evaluating quality of marquetries by applying active IR thermography and advanced signal processing. J. Therm. Anal. Calorim..

[8] Garrido I., Lagüela S., Sfarra S., Arias P. (2020). Development of Thermal Principles for the Automation of the Thermographic Monitoring of Cultural Heritage. Sensors.

[9] Garrido I., Solla M., Lagüela S., Fernández N. (2020). IRT and GPR Techniques for Moisture Detection and Characterisation in Buildings. Sensors.

[10] Garrido I., Lagüela S., Otero R., Arias P. (2020). Thermographic methodologies used in infrastructure inspection: A review—data acquisition procedures. Infrared Phys. Technol..

[11] Garrido I., Lagüela S., Otero R., Arias P. (2020). Thermographic methodologies used in infrastructure inspection: A review—Post-processing procedures. Appl. Energy.

[12] Tattersall G.J. (2016). Infrared thermography: A non-invasive window into thermal physiology. Comp. Biochem. Physiol. Part A Mol. Integr. Physiol..

[13] Sfarra S., Cicone A., Yousefi B., Ibarra-Castanedo C., Perilli S., Maldague X. (2019). Improving the detection of thermal bridges in buildings via on-site infrared thermography: The potentialities of innovative mathematical tools. Energy Build..

[14] Meola C., Di Maio R., Roberti N., Carlomagno G.M. (2005). Application of infrared thermography and geophysical methods for defect detection in architectural structures. Eng. Fail. Anal..

[15] Solla M., Lagüela S., Fernández N., Garrido I. (2019). Assessing rebar corrosion through the combination of nondestructive GPR and IRT methodologies. Remote Sens..

[16] Lerma C., Barreira E., Almeida R.M.S.F. (2018). A discussion concerning active infrared thermography in the evaluation of buildings air infiltration. Energy Build..

[17] Bagavathiappan S., Lahiri B.B., Saravanan T., Philip J., Jayakumar T. (2013). Infrared thermography for condition monitoring—A review. Infrared Phys. Technol..

[18] Garrido I., Lagüela S., Sfarra S., Madruga F.J., Arias P. (2019). Automatic detection of moistures in different construction materials from thermographic images. J. Therm. Anal. Calorim..

[19] Baldinelli G., Bianchi F., Rotili A., Costarelli D., Seracini M., Vinti G., Asdrubali F., Evangelisti L. (2018). A model for the improvement of thermal bridges quantitative assessment by infrared thermography. Appl. Energy.

[20] Khodayar F., Sojasi S., Maldague X. (2016). IRT for NDT: 2050 Horizon. Quant. Infrared Thermogr. J..

[21] Ibarra-Castanedo C., Piau J.M., Guilbert S., Avdelidis N., Genest M., Bendada A., Maldague X.P.V. (2009). Comparative study of active thermography techniques for the nondestructive evaluation of honeycomb structures. Res. Nondestruct. Eval..

[22] Ibarra-Castanedo C., González D., Klein M., Pilla M., Vallerand S., Maldague X. (2004). Infrared image processing and data analysis. Infrared Phys. Technol..

[23] Yao Y., Sfarra S., Lagüela S., Ibarra-Castanedo C., Wu J.-Y., Maldague X.P.V., Ambrosini D. (2018). Active thermography testing and data analysis for the state of conservation of panel paintings. Int. J. Therm. Sci..

[24] Zhang H., Sfarra S., Saluja K., Peeters J., Fleuret J., Duan Y., Fernandes H., Avdelidis N., Ibarra-Castanedo C., Maldague X. (2017). Non-destructive Investigation of Paintings on Canvas by Continuous Wave Terahertz Imaging and Flash Thermography. J. Nondestruct. Eval..

[25] Ibarra-Castanedo C., Khodayar F., Klein M., Sfarra S., Maldague X., Helal H., Tayoubi M., Marini B., Barré J.C. (2017). Infrared vision for artwork and cultural heritage NDE studies: Principles and case studies. Insight Non-Destructive Test. Cond. Monit..

[26] Sfarra S., Fernandes H.C., López F., Ibarra-Castanedo C., Zhang H., Maldague X. (2018). Qualitative Assessments via Infrared Vision of Sub-surface Defects Present Beneath Decorative Surface Coatings. Int. J. Thermophys..

[27] Thickett D., Cheung C.S., Liang H., Twydle J., Gr Maev R., Gavrilov D. (2017). Using non-invasive non-destructive techniques to monitor cultural heritage objects. Insight Non-Destructive Test. Cond. Monit..

[28] Vavilov V., Marinetti S., Grinzato E., Bison P., Dal Toè S., Burleigh D. (2002). Infrared thermographic nondestructive testing of frescos: Thermal modeling and image processing of three dimensional heat diffusion phenomena. Mater. Eval..

[29] Garrido I., Lagüela S., Sfarra S., Solla M. (2019). Algorithms for the automatic detection and characterization of pathologies in heritage elements from thermographic images. International Archives of the Photogrammetry, Remote Sensing and Spatial Information Sciences-ISPRS Archives.

[30] Garrido I., Lagüela S., Sfarra S., Zhang H., Maldague X.P.V. (2019). Automatic Detection and Delimitation of Internal Moisture in Mosaics from Thermographic Sequences. Experimental Tests. Proceedings.

[31] Goodfellow I., Bengio Y., Courville A. (2016). Deep Learning.

[32] Merkel G., Povinelli R., Brown R. (2018). Short-Term Load Forecasting of Natural Gas with Deep Neural Network Regression. Energies.

[33] Wang S., Chen W., Xie S.M., Azzari G., Lobell D.B. (2020). Weakly Supervised Deep Learning for Segmentation of Remote Sensing Imagery. Remote Sens..

[34] Wang J., Zhu X., Gong S., Li W. Transferable Joint Attribute-Identity Deep Learning for Unsupervised Person Re-Identification. Proceedings of the IEEE Conference on Computer Vision and Pattern Recognition (CVPR).

[35] Nunez I., Marani A., Nehdi M.L. (2020). Mixture Optimization of Recycled Aggregate Concrete Using Hybrid Machine Learning Model. Materials.

[36] Mahdavinejad M.S., Rezvan M., Barekatain M., Adibi P., Barnaghi P., Sheth A.P. (2018). Machine learning for internet of things data analysis: A survey. Digit. Commun. Netw..

[37] Pack Kaelbling L., Littman M.L., Moore A.W., Hall S. (1996). Reinforcement Learning: A Survey. J. Artiicial Intell. Res..

[38] Abiodun O.I., Jantan A., Omolara A.E., Dada K.V., Mohamed N.A.E., Arshad H. (2018). State-of-the-art in artificial neural network applications: A survey. Heliyon.

[39] Kourou K., Exarchos T.P., Exarchos K.P., Karamouzis M.V., Fotiadis D.I. (2015). Machine learning applications in cancer prognosis and prediction. Comput. Struct. Biotechnol. J..

[40] Mahony N.O., Campbell S., Carvalho A., Harapanahalli S., Velasco-Hernandez G., Krpalkova L., Riordan D., Walsh J. Deep Learning vs. Traditional Computer Vision. Proceedings of the Science and Information Conference.

[41] Ramesh V. (2017). A Review on Application of Deep Learning in Thermography. Int. J. Eng. Manag. Res..

[42] Denil M., Shakibi B., Dinh L., Ranzato M., de Freitas N. (2013). Predicting Parameters in Deep Learning. Adv. Neural Inf. Process. Syst..

[43] Rosasco L., De Vito E., Caponnetto A., Piana M., Verri A. (2004). Are Loss Functions All the Same?. Neural Comput..

[44] Dung C.V., Anh L.D. (2019). Autonomous concrete crack detection using deep fully convolutional neural network. Autom. Constr..

[45] Cha Y.J., Choi W., Büyüköztürk O. (2017). Deep Learning-Based Crack Damage Detection Using Convolutional Neural Networks. Comput. Civ. Infrastruct. Eng..

[46] Gibb S., La H.M., Louis S. A Genetic Algorithm for Convolutional Network Structure Optimization for Concrete Crack Detection. Proceedings of the 2018 IEEE Congress on Evolutionary Computation (CEC).

[47] Hatir M.E., Barstuğan M., İnce İ. (2020). Deep learning-based weathering type recognition in historical stone monuments. J. Cult. Herit..

[48] Llamas J., Lerones P.M., Medina R., Zalama E., Gómez-García-Bermejo J. (2017). Classification of Architectural Heritage Images Using Deep Learning Techniques. Appl. Sci..

[49] Pierdicca R., Paolanti M., Matrone F., Martini M., Morbidoni C., Malinverni E.S., Frontoni E., Lingua A.M. (2020). Point Cloud Semantic Segmentation Using a Deep Learning Framework for Cultural Heritage. Remote Sens..

[50] Hu J., Xu W., Gao B., Tian G., Wang Y., Wu Y., Yin Y., Chen J. (2018). Pattern Deep Region Learning for Crack Detection in Thermography Diagnosis System. Metals.

[51] Duan Y., Liu S., Hu C., Hu J., Zhang H., Yan Y., Tao N., Zhang C., Maldague X., Fang Q. (2019). Automated defect classification in infrared thermography based on a neural network. NDT E Int..

[52] Ali R., Cha Y.-J. (2019). Deep Learning-and Infrared Thermography-Based Subsurface Damage Detection in a Steel Bridge. Master’s Thesis.

[53] Yousefi B., Kalhor D., Usamentiaga R., Lei L., Castanedo C.I., Maldague X.P.V. Application of Deep Learning in Infrared Non-Destructive Testing. Proceedings of the 14th Quantitative InfraRed Thermography Conference.

[54] Bang H.T., Park S., Jeon H. (2020). Defect identification in composite materials via thermography and deep learning techniques. Compos. Struct..

[55] Luo Q., Gao B., Woo W.L., Yang Y. (2019). Temporal and spatial deep learning network for infrared thermal defect detection. NDT E Int..

[56] Fang Q., Nguyen B.D., Ibarra Castanedo C., Duan Y., Maldague X., Oswald-Tranta B., Zalameda J.N. (2020). Defects detection in infrared thermography by deep learning algorithm. Thermosense: Thermal Infrared Applications XLII.

[57] Cheng C., Shang Z., Shen Z. (2020). Automatic delamination segmentation for bridge deck based on encoder-decoder deep learning through UAV-based thermography. NDT E Int..

[58] Al-Habaibeh A., Sen A., Chilton J. (2020). Evaluation Tool For The Thermal Performance of Retrofitted Buildings Using An Integrated Approach of Deep Learning Artificial Neural Networks and Infrared Thermography. Energy Built Environ..

[59] Janssens O., Van De Walle R., Loccufier M., Van Hoecke S. (2018). Deep Learning for Infrared Thermal Image Based Machine Health Monitoring. IEEE/ASME Trans. Mechatron..

[60] Ornek A.H., Ceylan M. Comparison of traditional transformations for data augmentation in deep learning of medical thermography. Proceedings of the 42nd International Conference on Telecommunications and Signal Processing (TSP 2019).

[61] Garrido I., Lagüela S., Arias P., Balado J. (2018). Thermal-based analysis for the automatic detection and characterization of thermal bridges in buildings. Energy Build..

[62] He K., Gkioxari G., Dollár P., Girshick R. (2020). Mask R-CNN. IEEE Trans. Pattern Anal. Mach. Intell..

[63] Madruga F.J., Sfarra S., Perilli S., Pivarčiová E., López-Higuera J.M. (2020). Measuring the Water Content in Wood Using Step-Heating Thermography and Speckle Patterns-Preliminary Results. Sensors.

[64] Garrido I., Lagüela S., Arias P. Autonomous thermography: Towards the automatic detection and classification of building pathologies. Proceedings of the 14th Quantitative InfraRed Thermography Conference.

[65] Lin T.-Y., Maire M., Belongie S., Bourdev L., Girshick R., Hays J., Perona P., Ramanan D., Zitnick C.L., Dolí P. (2014). Microsoft COCO: Common Objects in Context. Comput. Sci..

[66] Ren S., He K., Girshick R., Sun J. (2017). Faster R-CNN: Towards Real-Time Object Detection with Region Proposal Networks. IEEE Trans. Pattern Anal. Mach. Intell..

[67] Long J., Shelhamer E., Darrell T. (2014). Fully Convolutional Networks for Semantic Segmentation. IEEE Trans. Pattern Anal. Mach. Intell..

[68] Zaniolo L., Marques O. (2020). On the use of variable stride in convolutional neural networks. Multimed. Tools Appl..

[69] Dumoulin V., Visin F. A Guide to Convolution Arithmetic for Deep Learning. http://arxiv.org/abs/1603.07285.

[70] Convolutional Neural Networks–Cezanne Camacho–Machine and Deep Learning Educator. https://cezannec.github.io/Convolutional_Neural_Networks/.

[71] Dahl G.E., Sainath T.N., Hinton G.E. Improving deep neural networks for LVCSR using rectified linear units and dropout. Proceedings of the ICASSP, IEEE International Conference on Acoustics, Speech and Signal Processing-Proceedings.

[72] Giusti A., Cireşan D.C., Masci J., Gambardella L.M., Schmidhuber J. Fast image scanning with deep max-pooling convolutional neural networks. Proceedings of the 2013 IEEE International Conference on Image Processing, ICIP 2013-Proceedings.

[73] Jo Y., Oh S.W., Kang J., Kim S.J. Deep Video Super-Resolution Network Using Dynamic Upsampling Filters Without Explicit Motion Compensation. Proceedings of the IEEE Computer Society Conference on Computer Vision and Pattern Recognition, IEEE Computer Society.

[74] Convolutional Neural Networks: The Theory-Bouvet Norge. https://www.bouvet.no/bouvet-deler/understanding-convolutional-neural-networks-part-1.

[75] How to use the UpSampling2D and Conv2DTranspose Layers in Keras. https://machinelearningmastery.com/upsampling-and-transpose-convolution-layers-for-generative-adversarial-networks/.

[76] Basha S.H.S., Dubey S.R., Pulabaigari V., Mukherjee S. (2020). Impact of fully connected layers on performance of convolutional neural networks for image classification. Neurocomputing.

[77] Kouretas I., Paliouras V. Simplified Hardware Implementation of the Softmax Activation Function. Proceedings of the 2019 8th International Conference on Modern Circuits and Systems Technologies, MOCAST 2019.

[78] Arunava Convolutional Neural Network. https://towardsdatascience.com/convolutional-neural-network-17fb77e76c05.

[79] Mask_RCNN/model.py at Master ·matterport/Mask_RCNN GitHub. https://github.com/matterport/Mask_RCNN/blob/master/mrcnn/model.py.

[80] Hosang J., Benenson R., Schiele B. Learning non-maximum suppression. Proceedings of the 30th IEEE Conference on Computer Vision and Pattern Recognition (CVPR 2017).

[81] He K., Zhang X., Ren S., Sun J. Deep Residual Learning for Image Recognition. Proceedings of the IEEE Computer Society Conference on Computer Vision and Pattern Recognition, IEEE Computer Society.

[82] Lin T.-Y., Dollár P., Girshick R., He K., Hariharan B., Belongie S. Feature Pyramid Networks for Object Detection. Proceedings of the 30th IEEE Conference on Computer Vision and Pattern Recognition, CVPR 2017.

[83] Firiuza ROI Pooling vs. ROI Align. https://medium.com/@Firiuza/roi-pooling-vs-roi-align-65293ab741db.

[84] Dutta A., Zisserman A. The VIA Annotation Software for Images, Audio and Video. MM 2019-Proc. Proceedings of the 27th ACM International Conference on Multimedia.

[85] Sfarra S., Theodorakeas P., Černecký J., Pivarčiová E., Perilli S., Koui M. (2017). Inspecting Marquetries at Different Wavelengths: The Preliminary Numerical Approach as Aid for a Wide-Range of Non-destructive Tests. J. Nondestruct. Eval..

[86] Usamentiaga R., Venegas P., Guerediaga J., Vega L., Molleda J., Bulnes F. (2014). Infrared Thermography for Temperature Measurement and Non-Destructive Testing. Sensors.

[87] Schwarz K., Heitkötter J., Heil J., Marschner B., Stumpe B. (2018). The potential of active and passive infrared thermography for identifying dynamics of soil moisture and microbial activity at high spatial and temporal resolution. Geoderma.

[88] Choi M., Kang K., Park J., Kim W., Kim K. (2008). Quantitative determination of a subsurface defect of reference specimen by lock-in infrared thermography. NDT E Int..

[89] Erazo-Aux J., Loaiza-Correa H., Restrepo-Giron A.D., Alfonso-Morales W. (2020). Optimized Gaussian model for non-uniform heating compensation in pulsed thermography. Appl. Opt..

[90] Usamentiaga R., Ibarra-Castanedo C., Maldague X. (2018). More than Fifty Shades of Grey: Quantitative Characterization of Defects and Interpretation Using SNR and CNR. J. Nondestruct. Eval..

[91] Mustapha A., Mohamed L., Ali K. (2020). An Overview of Gradient Descent Algorithm Optimization in Machine Learning: Application in the Ophthalmology Field. Communications in Computer and Information Science.

[92] Senior A., Heigold G., Ranzato M., Yang K. An empirical study of learning rates in deep neural networks for speech recognition. Proceedings of the ICASSP, IEEE International Conference on Acoustics, Speech and Signal Processing-Proceedings.

[93] Sutskever I., Martens J., Dahl G., Hinton G. On the importance of initialization and momentum in deep learning. Proceedings of the 30th International Conference on Machine Learning, (PMLR, 2013).

[94] Zhang G., Wang C., Xu B., Grosse R. (2018). Three Mechanisms of Weight Decay Regularization. arXiv.

[95] Perez L., Wang J. (2017). The Effectiveness of Data Augmentation in Image Classification using Deep Learning. arXiv.

[96] Tan C., Sun F., Kong T., Zhang W., Yang C., Liu C. (2018). A survey on deep transfer learning. Lecture Notes in Computer Science (including subseries Lecture Notes in Artificial Intelligence and Lecture Notes in Bioinformatics).

[97] Mask_RCNN/samples/balloon at Master Matterport/Mask_RCNN GitHub. https://github.com/matterport/Mask_RCNN/tree/master/samples/balloon.

[98] He K., Lu Y., Sclaroff S. Local Descriptors Optimized for Average Precision. Proceedings of the IEEE Computer Society Conference on Computer Vision and Pattern Recognition, IEEE Computer Society.

